# Nuclear Export Inhibitor Selinexor Enhances Oncolytic Myxoma Virus Therapy against Cancer

**DOI:** 10.1158/2767-9764.CRC-22-0483

**Published:** 2023-06-01

**Authors:** Masmudur M. Rahman, Fleur van Oosterom, Junior A. Enow, Maksuda Hossain, Ami D. Gutierrez-Jensen, Mackenzie Cashen, Anne Everts, Kenneth Lowe, Jacquelyn Kilbourne, Juliane Daggett-Vondras, Timothy L. Karr, Grant McFadden

**Affiliations:** 1 Center for Immunotherapy, Vaccines, and Virotherapy, Biodesign Institute, Arizona State University, Tempe, Arizona.; 2 School of Life Sciences, Arizona State University, Tempe, Arizona.

## Abstract

Oncolytic viruses exploited for cancer therapy have been developed to selectively infect, replicate, and kill cancer cells to inhibit tumor growth. However, in some cancer cells, oncolytic viruses are often limited in completing their full replication cycle, forming progeny virions, and/or spreading in the tumor bed because of the heterogeneous cell types within the tumor bed. Here, we report that the nuclear export pathway regulates oncolytic myxoma virus (MYXV) infection and cytoplasmic viral replication in a subclass of human cancer cell types where viral replication is restricted. Inhibition of the XPO-1 (exportin 1) nuclear export pathway with nuclear export inhibitors can overcome this restriction by trapping restriction factors in the nucleus and allow significantly enhanced viral replication and killing of cancer cells. Furthermore, knockdown of XPO-1 significantly enhanced MYXV replication in restrictive human cancer cells and reduced the formation of antiviral granules associated with RNA helicase DHX9. Both *in vitro* and *in vivo*, we demonstrated that the approved XPO1 inhibitor drug selinexor enhances the replication of MYXV and kills diverse human cancer cells. In a xenograft tumor model in NSG mice, combination therapy with selinexor plus MYXV significantly reduced the tumor burden and enhanced the survival of animals. In addition, we performed global-scale proteomic analysis of nuclear and cytosolic proteins in human cancer cells to identify the host and viral proteins that were upregulated or downregulated by different treatments. These results indicate, for the first time, that selinexor in combination with oncolytic MYXV can be used as a potential new therapy.

**Significance:**

We demonstrated that a combination of nuclear export inhibitor selinexor and oncolytic MYXV significantly enhanced viral replication, reduced cancer cell proliferation, reduced tumor burden, and enhanced the overall survival of animals. Thus, selinexor and oncolytic MYXV can be used as potential new anticancer therapy.

## Introduction

Oncolytic viruses (OV) have emerged as novel anticancer immunotherapies for treating standard therapy–resistant and metastatic cancers (1–3). An ideal replication-competent OV is expected to selectively infect, replicate, and generate progeny virions in infected cancer cells, which subsequently infect neighboring cancer cells in the tumor bed (4, 5). This successful replication of OVs is thought to mediate antitumoral activity in multiple ways, such as direct killing of infected cancer cells, exposing and presenting novel tumor-specific neoantigens, activation of systemic antitumor and antiviral immunity, and recruitment of activated immune cells to the tumor microenvironment (6, 7). In addition to their own multi-mechanistic antitumor activity, OVs can be combined with most currently approved cancer therapeutics, such as chemotherapies, immune checkpoint inhibitors, and cell therapies, for additional therapeutic benefits (8–11).

Myxoma virus (MYXV) has been developed as an OV against diverse malignancies (12, 13). MYXV is a prototypic member of the Leporipoxvirus genus in the Poxviridae family of viruses. Different isolates of MYXV cause disease only in European rabbits but are completely nonpathogenic to all other non-leporid species, including mice and humans. However, MYXV can productively infect many (but not all) classes of human cancer cells originating from different tissues, both *in vitro* and *in vivo*. This natural and selective tropism of MYXV for cancer cells and tissues allows its exploitation as an oncolytic virotherapeutic in several preclinical cancer models for various cancer types, such as pancreatic cancer, lung cancer, glioblastoma, ovarian cancer, melanoma, and hematologic malignancies (12–14).

Similar to other poxviruses, MYXV can promiscuously bind, enter, and initiate infection in a broad diversity of cancerous and noncancerous cells from most vertebrate species. However, the ability of MYXV to productively replicate and produce progeny in any cell type outside the rabbit largely depends on whether the virus can successfully overcome diverse intrinsic and innate antiviral cellular barriers (15). These barriers are sufficiently robust to restrict MYXV replication post-entry in normal primary somatic human or mouse cells, but tend to become compromised when cells are immortalized, transformed, or cancerous. Thus, unlike rabbit cells, where MYXV can counteract every aspect of these cellular barriers, in non-rabbit normal cells and a subset of cancer cells, complete replication of MYXV can be restricted to different levels by multiple factors. In human cancer cells, activation of these intrinsic cellular restriction factors and virus-induced signaling pathways can limit the replication and oncolytic ability of MYXV in specific cancer cell types, which we refer to as either nonpermissive or semipermissive. Several cellular pathways that are currently known to contribute to MYXV's ability of MYXV to replicate in human cancer cells include (i) endogenously activated protein kinase B (PKB)/AKT, (ii) antiviral pathway activated by protein kinase R, (iii) status of tumor suppressors such as p53, Rb, and ataxia telangiectasia, and (iv) antiviral states induced by IFNs or TNF (16–19). In addition to these cellular barriers, we recently reported that members of the cellular DEAD-box RNA helicase superfamily have potent antiviral and/or proviral functions that regulate MYXV replication in diverse human cancer cell types (20). Among these antiviral RNA helicases, we also reported that RNA helicase A (RHA) or DHX9 exits the nucleus in response to MYXV infection to form unique antiviral granules in the cytoplasm of infected human cancer cells. These antiviral granules are formed during the late replication phase of MYXV, which reduces MYXV late protein synthesis and limits MYXV replication and the generation of progeny virions (21). Furthermore, DHX9 knockdown significantly enhanced MYXV replication in both semipermissive and nonpermissive human cancer cell lines.

Here, we report that inhibition of the XPO1, also known as CRM1 (chromosome region maintenance 1) nuclear export pathway in diverse human cancer cell types where MYXV replication is restricted significantly enhances virus replication and progeny virus formation by reducing the appearance of cytoplasmic antiviral granules. The FDA-approved nuclear export inhibitor selinexor also significantly enhanced MYXV replication in diverse human cancer cells. A combination of selinexor and MYXV treatment significantly reduced cancer cell proliferation and enhanced cell death. Furthermore, using three-dimensional (3D) spheroid cultures of human cancer cells, we showed that selinexor enhanced MYXV replication and penetrative spread in spheroid cultures of cancer cells. We next tested human cancer cell-derived xenograft (CDX) models in NSG mice to determine the *in vivo* effect of selinexor on oncolytic MYXV replication. Similar to *in vitro* cultures, selinexor enhanced MYXV gene expression and replication in Colo205 and HT29 cell-derived CDX models in NSG mice. In addition, using PANC-1 cell-derived CDX models, we showed that selinexor plus MYXV treatment significantly reduced the tumor burden compared with the control or MYXV treatments. Furthermore, selinexor plus MYXV treatment significantly enhanced the survival of the mice. These results suggest that selinexor and the oncolytic MYXV can be developed as a novel combination therapy for cancer.

## Materials and Methods

### Cells and Viruses

RK13 (catalog no. CCL-37), Vero (catalog no. CCL-81), A549 (catalog no. CCL-185), PANC-1 (catalog no. CRL-1469), MDA-MB435 (catalog no. HTB-129), HT29 (catalog no. HTB-38), and Colo205 (catalog no. CCL-222) cells were purchased from the ATCC. Individual cell lines were tested for *Mycoplasma* contamination every month using a universal *Mycoplasma* detection kit (catalog no. 30-1012K) from ATCC. Cells were authenticated by examination of morphology and consistent *in vitro* proliferation. RK13, Vero, A549, PANC-1, and MDA-MB435 cells were cultured in DMEM (Cytiva) supplemented with 10% FBS (Gibco), 2 mmol/L glutamine (Invitrogen), and 100 μg of penicillin-streptomycin (P/S; Invitrogen). HT29 and Colo205 cells were cultured in McCoy's 5 (Cytiva) and RPMI1640 (Cytiva) media, respectively, supplemented with 10% FBS, 2 mmol/L glutamine, and 100 μg of P/S. All the cultures were maintained at 37°C in a humidified 5% incubator. Individual cryovials were thawed and cells were grown no more than 20 passages.

### Reagents and Antibodies

Rabbit polyclonal antibodies against DHX9 and CRM1 and mouse mAb against β-actin were purchased from Thermo Fisher Scientific. Horseradish peroxidase–conjugated goat anti-rabbit and anti-mouse IgG antibodies were purchased from Jackson Immuno Research Laboratories. All the secondary antibodies conjugated to Alexa Fluor 488, 594, 568, and 647 were purchased from Thermo Fisher Scientific. Selinexor (KPT330) was purchased from Apex Bio. The nuclear export inhibitors Leptomycin A and Leptomycin B (LMB), Ratjadone A, and Anguinomycin A purchased from Santa Cruz Biotechnology.

### Viruses and Viral Replication Assay

Wild-type myxoma virus constructs vMyx-GFP [WT-MYXV that expresses GFP under a poxvirus synthetic early/late promoter (sE/L)], vMyx-GFP-TdTomato (WT-MYXV, which expresses GFP under a poxvirus sE/L promoter and TdTomato under a poxvirus p11 late promoter), vMyx-FLuc (WT-MYXV that expresses firefly luciferase under a poxvirus sE/L promoter and TdTomato under a poxvirus p11 late promoter), and myxoma virus lacking the M11 L gene (vMyx-M11L-KO) were used (21, 22). All the myxoma viruses were grown in Vero cells. The virus stocks used were prepared using sucrose gradient purification as described previously (23).

Viral titers in different human cancer cell lines were determined using a viral replication assay. The cells were seeded in 24-well plate (2 × 10^5^ cells/well). The next day, the cells were treated with different concentrations of LMB or selinexor diluted in DMEM for 1 hour. MYXV was added to the cells and incubated for 1 hour at 37°C. After 1 hour, the unbound virus was washed away using DMEM, and DMEM with LMB or selinexor was added to the cells again. Cells were harvested in DMEM without LMB or selinexor at the indicated timepoints. After harvesting the cells, they were stored in a −80°C freezer until processing. Samples were subjected to three freeze/thaw cycles and 1-minute sonification to lyse the cells and release the viral particles. Afterward, different dilutions were prepared in DMEM and plated on rabbit RK13, and foci were counted after 48 hours using a fluorescent microscope. All assays and dilutions were performed in triplicates.

### Spheroid Generation and Virus Infection

Different cancer cell lines were grown and maintained as described in the cell culture section, and used for spheroid generation within 2–5 passages. A total of 96-well plates were prepared with Rat tail collagen I (Gibco) to form the surface for spheroid culture. On the day of cell seeding for spheroid generation, the cells were dissociated with TrypLE reagent (Gibco), neutralized the TrypLE reagent with fresh complete media, spun down, and resuspended in fresh complete media. After making a single-cell suspension, cells were counted using a Countess II automated cell counter and 1,000 cells in 100 μL volume were plated on the surface of the collagen matrix. The cells were observed daily for spheroid formation. After 5–7 days, when the spheroids reached the desired size, they were treated with selinexor and then infected with the vMyx-GFP-TdTomato virus.

### Immunofluorescence

Cells (5 × 10^5^–1 × 10^6^/dish) were seeded onto glass bottom 35 mm petri dishes overnight. Depending on the experiment, the next day, cells were transfected with siRNA for 48 hours or treated with the nuclear export inhibitor, MYXV, or a combination of both. At different timepoints after treatment, the cells were washed with PBS three times, fixed with 2% paraformaldehyde (Sigma-Aldrich) in PBS for 12 minutes at room temperature, washed with PBS three times, and permeabilized in 0.1% Triton X-100 (Sigma-Aldrich) in PBS for 90 seconds at room temperature. Fixed cells were washed with PBS three times and then blocked with 3% BSA (Sigma-Aldrich) in PBS for 30 minutes at 37°C. Samples were then incubated with primary antibody (1:300 dilution) for 30 minutes at 37°C, washed with PBS six times, and incubated with secondary antibodies conjugated to different Alexa Fluor. After washing again with PBS six times, samples were mounted on glass slides with Vecta Shield (Vectorlabs) containing DAPI (4′,6-diamidino-2-phenylindole) to stain DNA in the nuclei and viral factory. Images were captured using a fluorescence microscope (Leica).

### siRNA Transfection

ON-TARGETplus SMART pool siRNAs for CRM1/XPO1 and a non-targeting control (NT siRNA) were purchased from Dharmacon (Horizon Discovery). In 24-well plate, cells were seeded with 40%–50% confluence, left overnight for adherence, and then transfected with siRNAs (50 nmol/L) using Lipofectamine RNAiMAX (Invitrogen) transfection reagent. After 48 hours of transfection, the cells were infected with different multiplicity of infection (MOI) of vMyx-GFP for 1 hour, washed to remove the unbound virus, and incubated with complete media. At the indicated timepoints, cells were either observed under a fluorescence microscope to monitor and record the expression of fluorescent proteins or harvested and processed for titration of progeny virions.

### Click-iT EdU Cell Proliferation Assay

To visualize and measure cell proliferation, a Click-iT EdU cell proliferation assay (Thermo Fisher Scientific) was performed according to the manufacturer's instructions. Briefly, cells (5 × 10^5^/dish) were seeded on glass-bottom dishes and allowed to adhere by incubation overnight at 37°C. The next day, the cells were treated with selinexor, MYXV, or a combination of both for 24 hours. Subsequently, 5-ethynyl-2′-deoxyuridine (EdU) reagent (10 μmol/L) was added, and the cells were incubated for another 24 hours. To visualize EdU incorporation in dividing cells, the cells were fixed with 3.7% formaldehyde in PBS and permeabilized with 0.5% Triton X-100 in PBS. Cells were then incubated with the Click-iT EdU reaction cocktail with Alexa-Fluor-594 for 30 minutes at room temperature and protected from light. The cells were washed with PBS and stained with Nuclear Mask Blue for nuclear staining. Fluorescence images were obtained using a fluorescence microscope, and fluorescence signals were analyzed using ImageJ software.

### Cell Proliferation Assay

To measure cancer cell proliferation based on the amount of cellular DNA, the CyQuant no freeze (NF) Cell Proliferation Assay Kit (Invitrogen) was used according to the manufacturer's instructions. Briefly, Panc-1, HT29, MDA MB 435, and Colo205 cells were seeded in a 96-well plate (1 × 10^4^ cells/well) and left to attach to the wells overnight. The next day, the medium was removed and replaced with 50 μL medium containing different concentrations of selinexor (0–1 μmol/L). After an hour incubation with selinexor, the virus was added to different MOIs (MOI 0.5 – MOI 5), bringing the end volume of every well up to 100 μL. A 1× dye binding solution was prepared by adding 9 μL of the CyQuant NF Dye reagent in 4.5 mL Hank's Balanced Salt Solution buffer (Invitrogen). After 24, 48, 72, and 96 hours of incubation, the medium was removed from the cells and 50 μL of the 1x dye solution was added to all wells. The microplate was covered to protect it from light and was incubated for 30–60 minutes in a humidified 5% CO_2_ incubator at 37°C. Subsequently, cell proliferation was quantified by measuring fluorescence with excitation at 485 nmol/L and emission detection at 530 nmol/L in the VarioSkan Lux Microplate reader (Thermo Fisher Scientific). All experiments were performed in quadruples and normalized to mock-treated cells.

### Cell Viability Assay

To assess the viability of different human cancer cells after selinexor treatment or MYXV infection, 10,000 cells were seeded into each well of a 96-well plate. The next day, cells were either treated with different concentrations of selinexor, infected with different MOIs of MYXV, or treated with different concentrations of selinexor for 1 hour followed by infection with different MOIs of MYXV. A minimum of four to five wells were used for each treatment condition, and untreated cells (mock) served as controls. Cell viability was such at 24, 48, 72, and 96 hours cell viability was assessed using MTS assay.

### Animal Studies

All animal experiments were approved by the Institutional Animal Care and Use Committee (IACUC) of Arizona State University (Tempe, AZ) and conformed to all regulatory standards. Male and female NSG mice were purchased from the Jackson laboratory at 6–8 weeks of age. After arrival, the animals were housed in the vivarium of the Biodesign Institute under sterile conditions. The animals were acclimatized for at least 7 days before tumor implantation or any experimental procedures. All animal handling, housing, husbandry, and experimental protocols were performed according to the approved IACUC protocols and institutional standards. Cells (1 × 10^6^/mouse in 100 μL PBS) were subcutaneously injected into the flanks of NSG mice. When the average tumor volumes reached 50–200 mm^3^, the mice were randomized into different treatment groups. Each treatment group contained 5 or 6 animals. Tumor volume was measured two to three times per week as follows: volume = ½ (length × width^2^). When the tumor volume reached 1.5–2.0 cm^3^, the animals were euthanized, and tumors were collected for histology or processed for virus titration. To detect MYXV replication in the tumor bed, luciferin was injected via intraperitoneal delivery, and bioluminescence images were taken (Xenogen IVIS 2000).

### Nucleus-Cytoplasmic Fractionation and Proteomics

Human colorectal cancer cell line Colo205 was collected 48 hours after treatment with selinexor, MYXV infection, or selinexor + MYXV, and nuclear and cytosolic fractions were prepared using NE-PER nuclear and cytoplasmic extraction reagents (Thermo Fisher Scientific). The purity of the fractions was confirmed by Western blot analysis of tubulin (cytoplasmic) and histone H3 (nuclear). These fractions were used for LC/MS analysis at the Biosciences Mass Spectrometry Core Facility at Arizona State University (Tempe, AZ). For LC/MS-MS, solubilized proteins were quantified (Thermo Fisher EZQ Protein Quantitation Kit or the Pierce BCA). Proteins were reduced with 50 mmol/L dithiothreitol (Sigma-Aldrich) at 95°C for 10 minutes and alkylated for 30 minutes with 40 mmol/L iodoacetamide (Pierce). Proteins were digested using 2.0 μg of mass spectrometry (MS)-grade porcine trypsin (Pierce) and peptides were recovered using S-trap Micro Columns (ProtiFi) per manufacturer directions. Recovered peptides were dried via speed vac and resuspended in 30 μL of 0.1% formic acid. All data-dependent mass spectra were collected in positive mode using an Orbitrap Fusion Lumos mass spectrometer (Thermo Fisher Scientific) coupled with an UltiMate 3000 UHPLC (Thermo Fisher Scientific). A total of 1 μL of the peptide was fractionated using an Easy-Spray LC column (50 cm Å–75 μm ID, PepMap C18, 2 μm particles, 100 Å pore size, Thermo Fisher Scientific) with an upstream 300 μm Å–5 mm trap column. Electrospray potential was set to 1.6 kV and the ion transfer tube temperature to 300°C. The mass spectra were collected using the “Universal” method optimized for peptide analysis provided by Thermo Fisher Scientific. Full MS scans (375–1,500 m/z range) were acquired in profile mode with the following settings: Orbitrap resolution 120,000 (at 200 m/z), cycle time 3 seconds and mass range “Normal;” RF lens at 30% and the AGC set to “Standard”; maximum ion accumulation set to “Auto”; monoisotopic peak determination (MIPS) at “peptide” and included charge states 2–7; dynamic exclusion at 60 seconds, mass tolerance 10 ppm, intensity threshold at 5.0e3; MS-MS spectra acquired in a centroid mode using quadrupole isolation at 1.6 (m/z); collision-induced fragmentation energy at 35%, activation time 10 ms. Spectra were acquired over a 240-minute gradient, flow rate 0.250 μL/minute as follows: 0–3 minutes at 2%, 3–75 minutes at 2%–15%, 75–180 minutes at 15%–30%, 180–220 minutes at 30%–35%, 220–225 minutes at 35%–80% 225–230 at 80% and 230–240 at 80–5%.

### Label-free Quantification

Raw spectra were loaded into Proteome Discover 2.4 (Thermo Fisher Scientific) and protein abundances were determined using UniProt (www.uniprot.org) *Homo sapiens* database (Hsap UP000005640.fasta). Protein abundances were determined using raw files and were searched using the following parameters: Trypsin as an enzyme, maximum missed cleavage site 3, min/max peptide length 6/144, precursor ion (MS1) mass tolerance at 20 ppm, fragment mass tolerance at 0.5 Da, and a minimum of 1 peptide identified. Carbamidomethyl (C) was specified as fixed modification and dynamic modifications set to Acetyl and Met-loss at the N-terminus, and oxidation of Met. A concatenated target/decoy strategy and a FDR set to 1.0% were calculated using Percolator. Accurate mass and retention time of detected ions (features) using the Minora Feature Detector algorithm were then used to determine the AUC of the selected ion chromatograms of the aligned features across all runs and the relative abundances calculated. Differential abundances between treatments were determined using protein abundance ratio *t* tests (background based) as implemented in Proteome Discoverer 2.4.

### Statistical Analysis

Statistical analyses were performed using GraphPad Prism software. Values are represented as mean ± SD for at least three or four independent experiments. ANOVA and *t* test (when only two groups were compared) were used to determine the significance. Kaplan–Meier analysis of mouse survival was performed using GraphPad Prism software, and the log-rank (Mantel–Cox) test was performed to compare survival curves and perform statistical analyses. *P* values are reported as follows: nonsignificant (ns), *P* > 0.05; *, *P* < 0.05; **, *P* < 0.01; ***, *P* < 0.001; ****, *P* < 0.0001.

### Data Availability Statement

The data generated in this study are available upon request from the corresponding authors.

## Results

### Nuclear Export Inhibitors Enhance MYXV Replication in Restricted Human Cancer Cell Lines by Reducing the Formation of DHX9 Antiviral Granules

**FIGURE 1:**
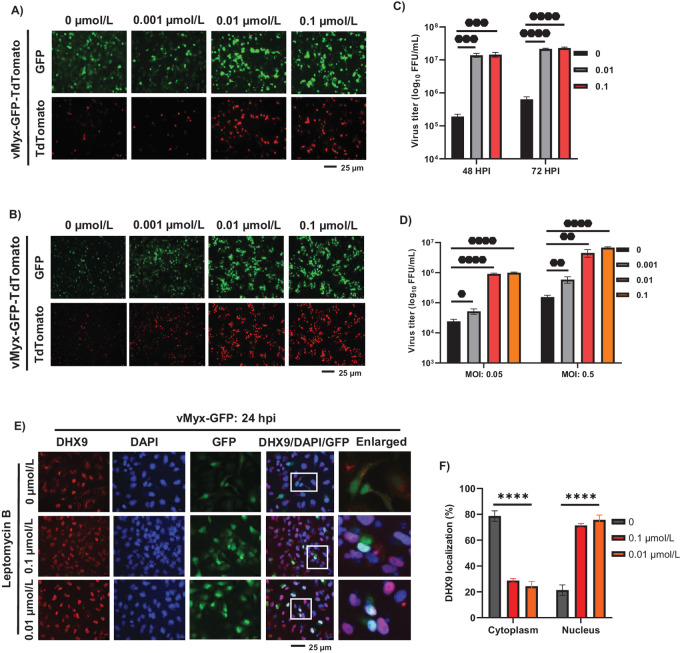
LMB treatment significantly enhanced MYXV replication in human cancer cells by reducing the formation of DHX9 antiviral granules. PANC-1 (**A**) and HT29 (**B**) cells were treated with different concentration of LMB for 1 hour, infected with different MOI of vMyx-GFP-TdTomato for 1 hour and replaced with fresh media containing the same doses of LMB. Fluorescence images were taken 48 hpi. PANC-1 (**C**) and HT29 (**D**) cells were harvested at different timepoints to determine progeny virus production by titration assays on permissive RK13 cells. Data represent mean ± SD and *n* = 3. Statistically significant differences in comparison with infection without LMB are indicated. **E,** A549 cells were treated with the indicated concentration of LMB for 1 hour and infected with vMyx-GFP (MOI = 1.0). After 24 hpi, the cells were fixed and stained with antibodies against DHX9. Nuclei were stained with DAPI and imaged by fluorescence microscope. **F,** Number of cells showing strong nuclear or cytoplasmic staining of DHX9 after 24 hpi. A minimum of 100 cells were used for analysis from fluorescence images taken in **E**. Data represent mean ± SD and *n* = 3. *, *P* < 0.05; **, *P* < 0.01; ***, *P* < 0.001; ****, *P* < 0.0001.

We previously reported that MYXV infection of human cancer cells results in the formation of cytosolic antiviral granules composed of RNA helicase DHX9 and reduced MYXV replication (21). Knockdown of DHX9 significantly enhances MYXV replication and progeny virus production in cancer cells, where viral replication is restricted. In uninfected cells, DHX9 was mainly localized in the nucleus; however, in MYXV-infected cells, DHX9 was detected in the cytoplasm associated with antiviral granules. Nuclear export and import pathways play major roles in the localization and function of many cellular proteins, such as RNA helicases (24, 25). Here, we tested whether nuclear export inhibitors that target the XPO1-mediated nuclear export pathway can block the formation of DHX9 antiviral granules in the cytoplasm. We initially examined the effect of LMB on MYXV replication in human cancer cells, such as PANC-1 (pancreatic cancer cell line) and HT29 (colorectal cancer cell line). In these cell lines, pretreatment with a lower concentration of LMB (between 0.1 and 0.001 μmol/L) that had reduced cytotoxicity significantly enhanced viral gene expression, as observed with increased early/late GFP and late TdTomato reporter proteins expression (Fig. 1A and B). This increased viral protein expression also significantly enhanced progeny virus production, as measured by the virus titration assay (Fig. 1C and D). We next checked whether this increase in viral gene expression and progeny virus formation correlated with the reduction or inhibition of the formation of DHX9 antiviral granules. Immunofluorescence staining of cells with anti-DHX9 antibody revealed that in most LMB plus MYXV-treated cells (>80%), DHX9 remained in the nucleus and blocked the formation of antiviral granules (Fig. 1E and F).

### Selinexor Enhances MYXV Replication in Restricted Human Cancer Cell Lines

**FIGURE 2:**
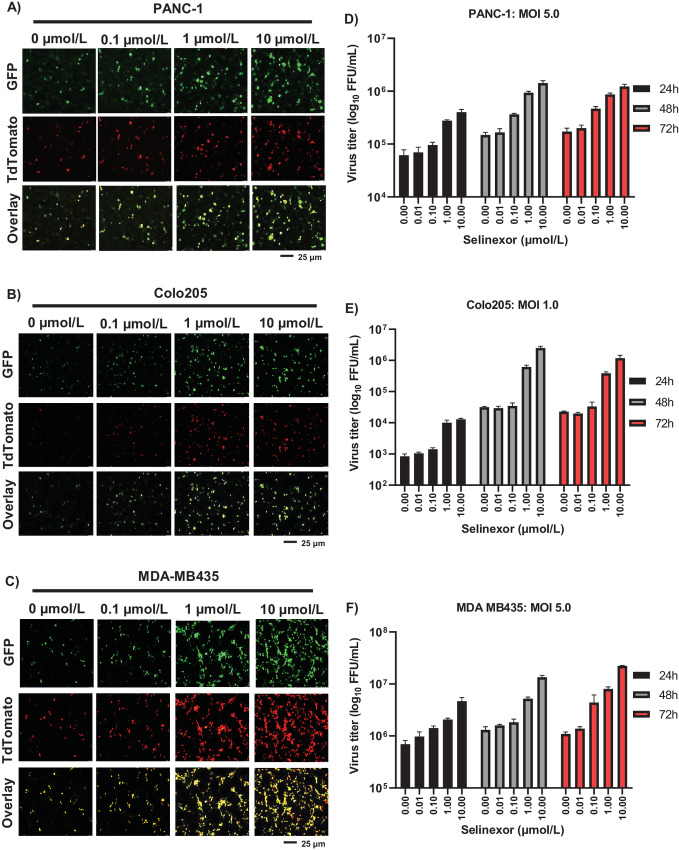
Selinexor treatment significantly enhanced MYXV gene expression and replication in diverse human cancer cell lines. PANC-1 (**A**), Colo205 (**B**), and MDA-MB435 (**C**) cells were treated with different concentration of selinexor for 1 hour, infected with different MOI of vMyx-GFP-TdTomato for 1 hour and replaced with fresh media containing the same doses of selinexor. Fluorescence images were taken 48 hpi. PANC-1 (**D**), Colo205 (**E**), and MDA-MB435 (**F**) cells were harvested at different times after infection to determine progeny virus production by titration assays on permissive RK13 cells. Data represent mean ± SD and *n* = 3. Statistically significant differences in comparison with infection without selinexor were observed.

The nuclear export inhibitor, selinexor, has been developed as a less toxic SINE compound that inhibit the XPO1/CRM1-mediated nuclear export pathway (26). Selinexor has been approved for use in patients with hematologic malignancies (27, 28). To assess whether selinexor enhances MYXV replication, similar to LMB, multiple MYXV-restricted human cancer cell lines, such as PANC-1 (Fig. 2A), Colo205 (Fig. 2B), and MDA-MB435 (Fig. 2C) were first treated with different concentrations of selinexor and infected with vMyx-GFP-TdTomato to monitor viral gene expression and replication. We also monitored and measured the cell viability (described in the next section). Between 10 and 0.01 μmol/L of selinexor pretreatment, we observed increased viral early/late GFP and late TdTomato reporter proteins expression in all the tested cell lines (Fig. 2A–C). Selinexor also enhanced virus spread and foci formation in these restricted human cancer cell lines when infected at a lower MOI. However, at a concentration of 10 μmol/L or higher, selinexor alone caused enhanced cell death in all cancer cell lines tested. We collected infected cells at different time points to further assess progeny virus formation and performed virus titration using permissive rabbit RK13 cells. In all the cell lines tested, we observed a significant increase (between 1 and 2 logs) in virus production compared with infection with MYXV alone (Fig. 2D–F). These results show that selinexor enhances MYXV gene expression, replication, and progeny virus formation in all tested cell lines where MYXV replication was restricted.

### CRM1/XPO1 Knockdown Enhances MYXV Replication in Restricted Human Cancer Cells and Reduces the Formation of DHX9-containing Cytoplasmic Antiviral Granules

**FIGURE 3:**
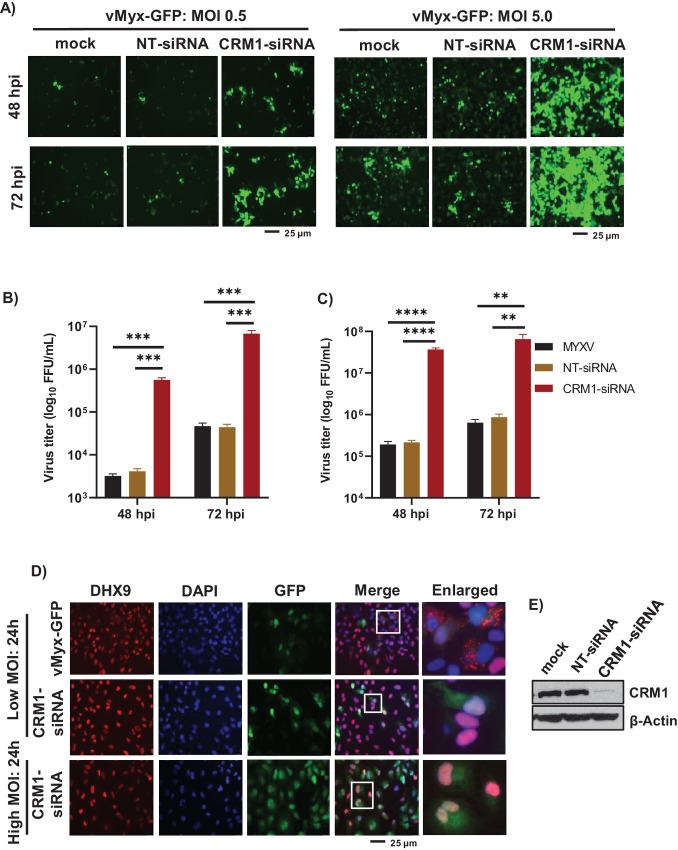
CRM1 knockdown significantly enhanced MYXV replication in human cancer cells. PANC-1 cells were transiently transfected with CRM1 siRNA or control NT siRNA. After 48 hours, the cells were infected with vMyx-GFP at MOI of 0.5 or 5.0 for 1 hour and replaced with fresh media. **A,** Images showing expression of GFP after 48 and 72 hpi. The cells were harvested at 48 and 72 hpi to determine progeny virus formation by titration assay on permissive RK13 cells. Virus titers after infection with an MOI of 0.5 (**B**) or 5.0 (**C**). Data represent mean ± SD and *n* = 3. Statistically significant differences in comparison with infection with virus alone are indicated. **, *P* < 0.01; ***, *P* < 0.001; ****, *P* < 0.0001. **D,** A549 cells were transfected with CRM1 siRNA and infected with vMyx-GFP [MOI = 0.1 (low MOI) or 3.0 (high MOI)]. After 24 hpi, the cells were fixed and stained with antibodies against DHX9. Nuclei were stained with DAPI and imaged by fluorescence microscope. **E,** Western blot analysis of CRM1 protein level in A549 cells after transfection of siRNAs for 48 hours; actin as loading control. Assays were performed in triplicate and a representative is shown.

Because inhibition of the CRM1/XPO1-mediated nuclear export pathway using selinexor or other SINEs reduced the formation of DHX9-containing antiviral granules and subsequently enhanced MYXV replication, we further extended this observation by direct knockdown of CRM1 using siRNA. After transfection of CRM1 siRNA or control siRNA (NT-siRNA) in PANC-1 cells, the cells were infected with vMyx-GFP at an MOI of 0.5 or 5.0. CRM1 protein knockdown using siRNA was confirmed by Western blot analysis (Fig. 3E). After CRM1 knockdown in PANC-1 cells, infection with an MOI of 0.5 allowed the formation of distinct larger foci compared with cells infected with the virus alone or NT siRNA (Fig. 3A, left top and bottom). Furthermore, infection of PANC-1 cells at an MOI of 5.0 resulted in significantly higher GFP expression in CRM1 knockdown cells, as observed under the microscope (Fig. 3A, right top and bottom). To measure progeny virus production, virus titrations were performed at 48 and 72 hpi. With both MOI of 0.5 or 5.0, we observed more than a 2 log increase in progeny virus titer (Fig. 3B and C). We then examined whether CRM1 knockdown reduced the formation of DHX9-containing antiviral granules in the cytoplasm after MYXV infection. Immunofluorescence microscopy results demonstrated that following CRM1 knockdown, DHX9 remained in the nucleus when the cells were infected with either low or high MOI and prevented the formation of DHX9-containing cytoplasmic antiviral granules (Fig. 3D). The retention of DHX9 in the nucleus also increased the number of GFP-positive virus-infected cells in the CRM1 siRNA-mediated knockdown cells, reflecting enhanced viral infection (Fig. 3D, GFP panels). These results confirm that the CRM1/XPO1 nuclear export pathway is mainly responsible for the transport of proteins that form antiviral granules and restrict MYXV replication in human cancer cells.

### The Combination of Selinexor and MYXV Reduces Cancer Cell Proliferation

**FIGURE 4:**
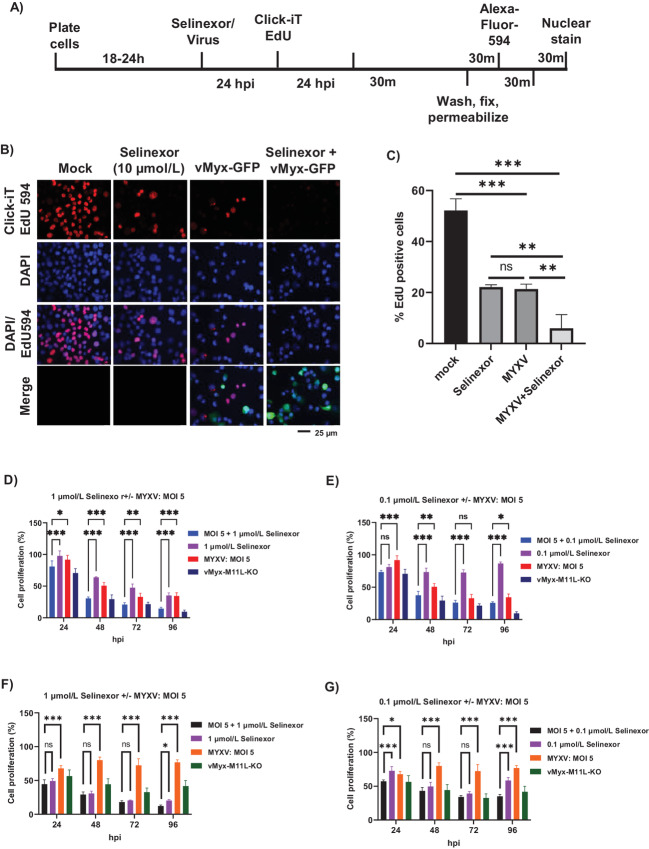
Combination of selinexor and oncolytic MYXV significantly reduced human cancer cells proliferation. **A,** Schematic representation of the cell proliferation assay protocol using Click-iT EdU kit. **B,** PANC-1 cells were left untreated (mock) or treated with selinexor, vMyx-GFP or a combination of selinexor + vMyx-GFP and cell proliferation assay was performed using Click-iT EdU kit. The images were taken using a fluorescence microscope. The assays were performed in triplicate and a representative is shown. **C,** Quantification of the number of cells showing labeling with EdU 594 (red fluorescence). A minimum of 100 cells were used for analysis from fluorescence images taken in **B**. Data represent mean ± SD and *n* = 3. Statistically significant differences among the different treatments are indicated. **, *P* < 0.01; ***, *P* < 0.001. PANC-1 (**D** and **E**) and Colo205 (**F** and **G**) cells in 96-well plates were left untreated (mock) or treated with selinexor, vMyx-GFP, a combination of selinexor + vMyx-GFP or vMyx-M11L-KO as control and cell proliferation assay was performed using CyQUANT NF cell proliferation assay kit. Data represent mean ± SD and *n* = 4. Statistically significant differences among the different treatments are indicated. ^ns^, *P* > 0.05; *, *P* < 0.05; **, *P* < 0.01; ***, *P* < 0.001.

Selinexor reduces the proliferation of cancer cells (29). However, viral infection also stops cell proliferation (30). We tested the effect of selinexor and MYXV on human cancer cells when treated alone or in combination. To assess this, we performed two cell proliferation assays. In the first method, we measured DNA synthesis by the incorporation of EdU, a nucleoside analog of thymidine, into DNA during active DNA synthesis (ref. 31; Fig. 4A). Using this method in uninfected PANC-1 cells, we detected EdU incorporation in more than 50% of dividing cells (Fig. 4B and C). When the cells were treated with selinexor or MYXV for 24 hours, we observed a significant reduction in cell proliferation (20% of cells were EdU-positive) compared with mock-treated cells. To observe the effect of the combination of selinexor and MYXV, we first treated the cells with selinexor for 1 hour and then infected them with MYXV for 24 hours in the presence of selinexor. This combination treatment further significantly reduced cell proliferation to single treatment and almost completely blocked EdU incorporation (Fig. 4B and C). To further confirm these observations, we performed another cell proliferation assay using CyQUANT, which measures the DNA content in the cells. Using this assay, we measured cell proliferation in PANC-1 (Fig. 4D and E) and Colo205 (Fig. 4F and G) cells at different timepoints after treatment with different concentrations of selinexor and multiple MOI of MYXV. In this assay, selinexor or MYXV alone significantly reduced cell proliferation after 24 hours compared with mock-treated cells. However, the combination of selinexor and MYXV considerably reduced cell proliferation compared with treatment with different concentrations of selinexor alone. As a control, we have used vMyx-M11L-KO, a MYXV construct lacking the expression of viral antiapoptotic protein M11 L (32). These results suggest that the combination of selinexor and MYXV infection almost entirely reduces cancer cell proliferation.

### The Combination of Selinexor and MYXV Reduces Cancer Cell Viability

**FIGURE 5:**
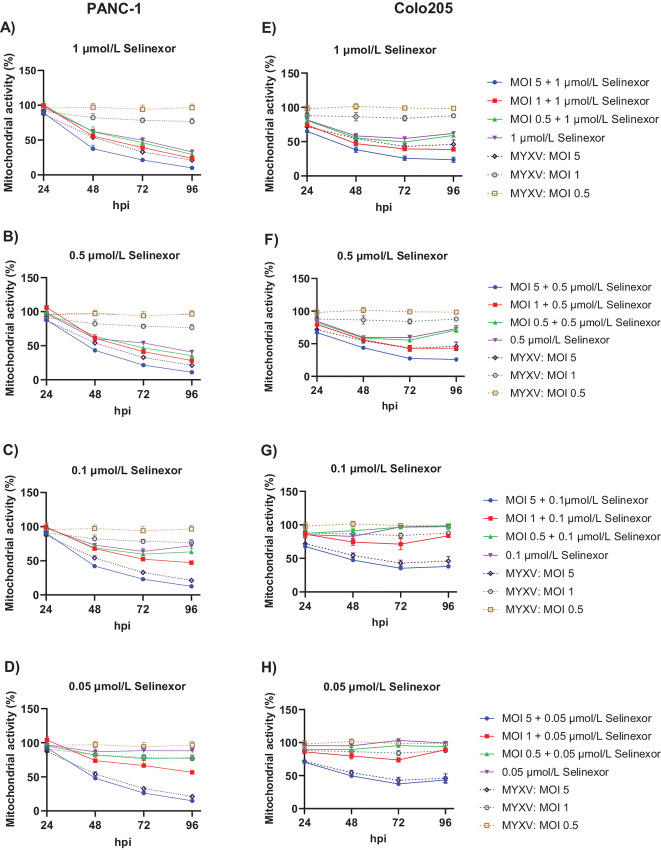
Combination of selinexor and oncolytic MYXV significantly reduced viability of human cancer cells. PANC-1 (**A–D**) and Colo205 (**E–H**) cells in 96-well plates were left untreated (mock) or treated with different concentration of selinexor, or infected with different MOI of MYXV, or treated with a combination of selinexor and MYXV for indicated timepoints. Cell viability was measured using the MTS assay reagents. Data represent mean ± SD and *n* = 4.

To further assess whether the inhibition of cell proliferation enhances cell death, we measured cell viability using an MTS assay to detect mitochondrial activity in active cells. For this assay, PANC-1 (Fig. 5A–D) and Colo205 (Fig. 5E–H) cells were treated with different concentrations of selinexor, infected with different MOI of MYXV, or treated with a combination of selinexor plus MYXV, and cell viability was measured at different timepoints. In all the tested cell lines, when treated with 1 or 0.5 μmol/L selinexor, cell viability reduced to almost 50% over time; however, at lower concentrations (0.1 and 0.05 μmol/L), selinexor had nearly no effect on cell viability. Similarly, infection with MYXV alone at an MOI of 5 significantly reduced cell viability (>50%) in almost all cell lines. However, MOI of 1.0, and 0.5, had practically no effect on cell viability in these nonpermissive cancer cells. As described in Figs. 1 and 2, with a lower MOI of MYXV infection and selinexor (0.1 μmol/L or lower) concentrations, we observed enhanced viral gene expression and replication. We then tested whether combining different concentrations of selinexor and MYXV infection at different MOI could further reduce cancer cell viability. Treatment with a combination of different concentrations of selinexor and infection with an MOI of 5 of MYXV showed significantly enhanced cell death compared with selinexor or MYXV treatment alone. We observed a significant reduction in cell viability with a combination of 0.5, 0.1, and 0.05 μmol/L selinexor and MOI 5 infection, where selinexor had almost no effect. Similarly, infection with a low MOI of 1 and 0.5, and a combination of 1 μmol/L selinexor significantly increased cell death in all tested cell lines. These results suggest that a concentration of selinexor that does not affect cell viability can enhance MYXV replication and significantly reduce cell viability by enhancing the oncolytic effect of MYXV.

### Selinexor Enhances MYXV Replication in 3D Human Cancer Cell Cultures

**FIGURE 6:**
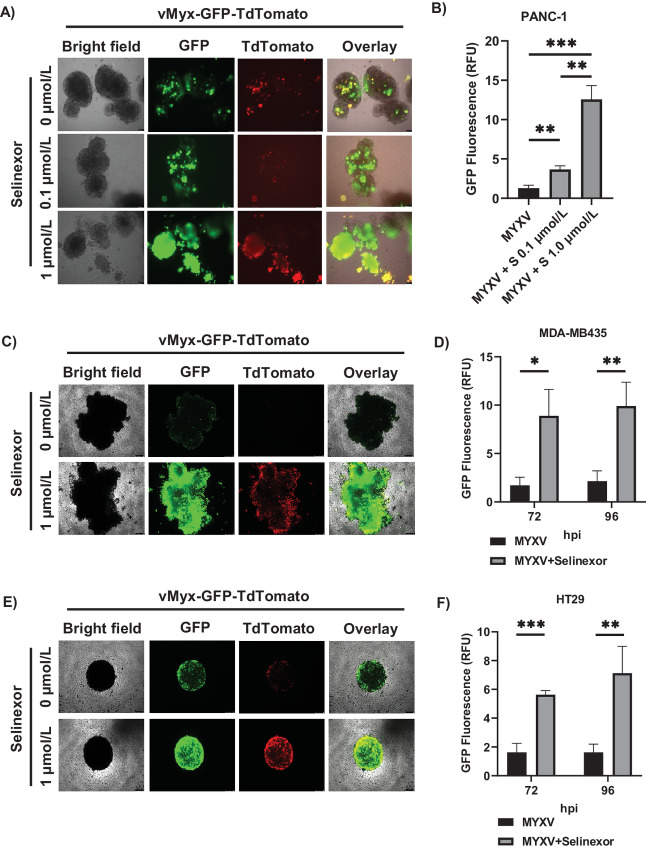
Selinexor significantly enhanced MYXV infection and replication in 3D culture of human cancer cell lines. PANC-1 (**A**), MDA-MB435 (**C**), and HT29 (**E**) cells were treated with different concentration of selinexor for 1 hour and then infected with vMyx-GFP-TdTomato in the presence of selinexor. Fluorescence images were taken 72 or 96 hpi. Quantification of the level of GFP fluorescence as relative fluorescence units (RFU) from PANC-1 (**B**), MDA-MB435 (**D**), and HT29 (**F**) cells are shown. Data represent mean ± SD and *n* = 3. Statistically significant differences in comparison with infection without selinexor are indicated. *, *P* < 0.05; **, *P* < 0.01; ***, *P* < 0.001.

*In vitro* 3D cell culture allows cells to contact and form a platform representing *in vivo* tumor mass (33). To test whether selinexor can enhance MYXV replication in 3D culture of human cancer cells, we established a 3D cell culture using type I collagen. We used various MYXV replication–restricted human cancer cell lines, including PANC-1 (Fig. 6A), HT29 (Fig. 6E), and MDA-MB435 (Fig. 6C) to form 3D spheroids. After forming 3D spheroids in each well of 96-well plates, the cells were individually treated with different concentrations of selinexor for 1 hour and then infected with vMyx-GFP-TdTomato in the presence of selinexor. Fluorescence microscopy images demonstrated that both GFP (early/late) and TdTomato (late) expression was enhanced in the selinexor-treated spheroids in all tested cell lines (Fig. 6A, C, and E). Quantification of GFP fluorescence using a plate reader also showed a significant increase in the level of GFP expression in selinexor-treated spheroids (Fig. 6B, D, and F). These results confirmed that selinexor enhances MYXV gene expression and replication in 3D spheroid human cell cultures. These findings prompted us to examine the effect of selinexor on MYXV replication *in vivo* in xenografted tumors in mice.

### Selinexor Enhances MYXV Replication *in vivo* in Xenografted Human Cancer Tumors and Reduces Tumor Burden

**FIGURE 7:**
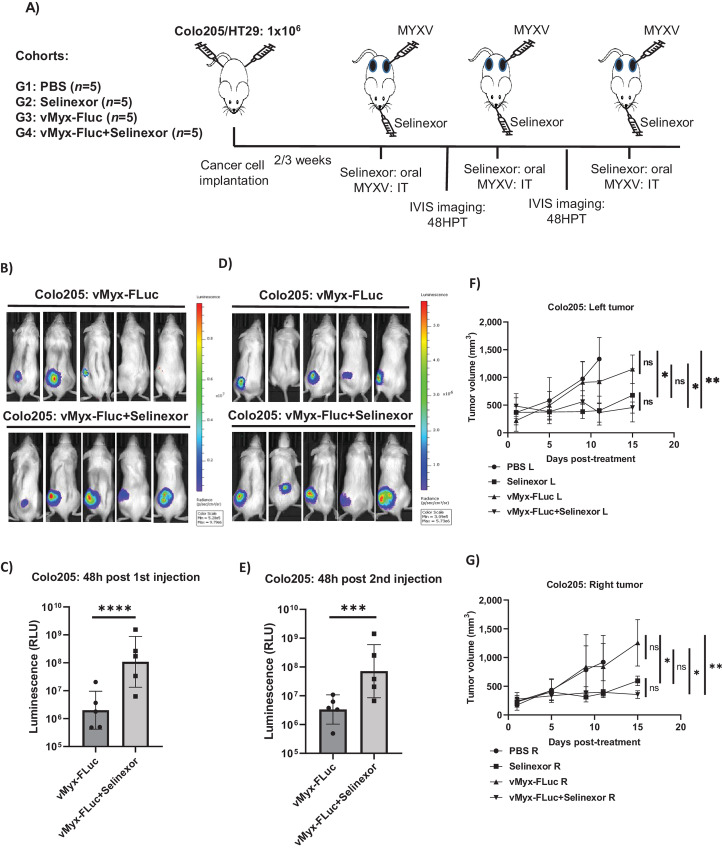
Selinexor enhances MYXV replication *in vivo* in xenograft tumors in NSG mice and reduces tumor burden. **A,** Diagram of experimental setup. NSG mice were inoculated with Colo205 or HT29 cells at day 0 via subcutaneous injection on both flanks. Animals received three doses of selinexor by oral gavage and three intratumoral injection of vMyx-FLuc on the left flank. Mice were imaged for luciferase activity 48 hours after first and second virus injection. **B,** Luminescence images taken using IVIS imaging system 48 hours after the first injection. **C,** Quantification of luminescence signals as relative light units (RLU) from images taken 48 hours after first injection. **D,** Luminescence images taken using IVIS imaging system 48 hours after the second injection. **E,** Quantification of luminescence signals as RLU from images taken 48 hours after second injection. **F**–**G,** Tumor volumes on the left and the right flanks were measured at different days after the start of treatments. Results are presented as mean ± SEM. ^ns^, *P* > 0.05; *, *P* < 0.05; **, *P* < 0.01.

To test whether selinexor can enhance MYXV replication *in vivo,* we established xenograft tumor models in immunodeficient NSG mice. Human Colo205 (Fig. 7) and HT29 (Supplementary Fig. S1) cells were injected subcutaneously on both sides of the flank to generate tumors (Fig. 7A; Supplementary Fig. S1A). After the tumor size reached approximately 100–200 mm^3^, the animals were randomly assigned to different treatment groups to maintain the average tumor size. Animals (*n* = 5) were treated with either selinexor alone (oral), vMyx-Fluc (WT MYXV expressing Firefly Luc and TdTomato) alone (intratumorally, 1 × 10^7^ FFU on the left tumor only), PBS (oral and intratumoral), or selinexor (oral) + vMyx-Fluc (intratumorally, 1 × 10^7^ FFU on the left tumor only). After 48 the post-first treatment, the mice were imaged using an IVIS system for luciferase expression. The images were analyzed, and the F-Luc signal was quantified using software. We observed that mice that received selinexor had significantly higher levels of luciferase signals than those injected with the virus alone (Fig. 7B). This enhanced luciferase signal was observed in both Colo205 (Fig. 7C) and HT29 recipient mice (Supplementary Fig. S1B). Subsequently, these mice received second and third dose of selinexor and an intratumoral injection of MYXV in the same tumor. IVIS imaging after the second treatment still showed a significantly enhanced level of luciferase signal from the virus in Colo205 tumors (Fig. 7D and E). In addition, we measured the tumor burden on both flanks during the course of the treatment (Fig. 7F and G). Treatment with selinexor alone and a combination of selinexor and MYXV significantly reduced tumor burden compared with PBS or MYXV-only treatments on both flanks in the Colo205 (Fig. 7F and G) and HT29 (Supplementary Fig. S1C and S1D) xenograft models. However, when comparing the size of selinexor and selinexor + MYXV-treated tumors, we observed no significant difference, although there was a trend that selinexor + MYXV-treated tumors were smaller in size than selinexor-only treatment (Supplementary Fig. S2). These results confirmed that selinexor enhanced MYXV gene expression and replication *in vivo*. In addition, selinexor alone or in combination with the oncolytic MYXV can reduce tumor burden in xenograft animal models.

### Selinexor in Combination with MYXV Reduces Tumor Burden and Extends the Survival of Animals in PANC-1 Xenograft Tumors

**FIGURE 8:**
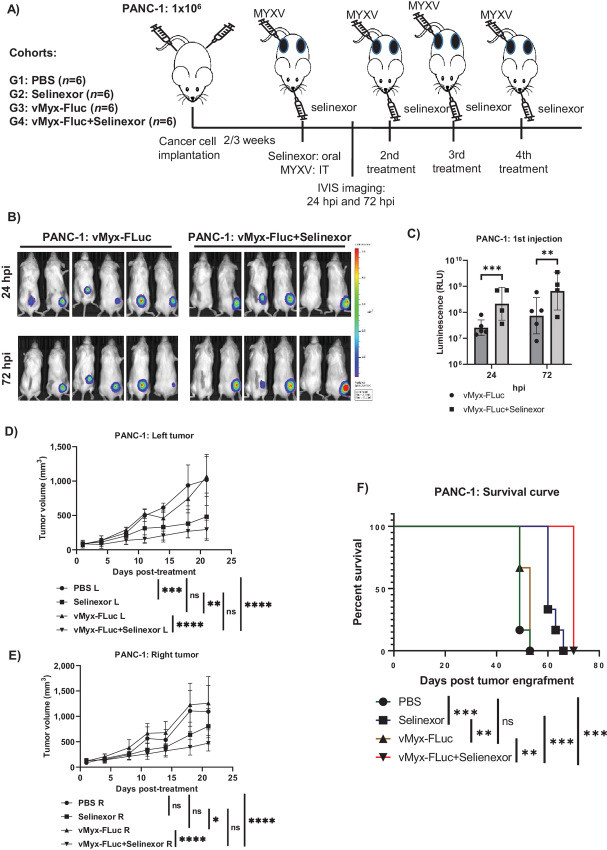
Selinexor significantly enhances MYXV replication in PANC-1 xenograft tumors in NSG mice, reduces tumor burden and prolong survival. **A,** Diagram of experimental setup. NSG mice were inoculated with PANC-1 cells at day 0 via subcutaneous injection on both flanks. Animals received four doses of selinexor by oral gavage and three intratumoral injection of vMyx-FLuc on the right flank. Mice were imaged for luciferase activity 24 and 48 hours after first virus injection. **B,** Luminescence images taken using IVIS imaging system 24 and 72 hours after the first injection. **C,** Quantification of luminescence signals as relative light units (RLU) from images taken 24 and 72 hours after first injection. **D**–**E,** Tumor volumes on the left and the right flanks were measured at different days after the start of treatments. Results are presented as mean ± SEM. **F,** Kaplan–Meier survival curves comparing animals with different treatment groups. ^ns^, *P* > 0.05; *, *P* < 0.05; **, *P* < 0.01; ***, *P* < 0.001; ****, *P* < 0.0001.

On the basis of our *in vivo* results showing that selinexor enhances MYXV replication in the tumor bed and reduces the tumor burden in Colo205 and HT29 xenograft tumors, we extended the study using PANC-1 cell xenograft tumors. Human PANC-1 cells were injected subcutaneously on both sides of the flank to generate tumors (Fig. 8A). After the tumor size reached approximately 50–100 mm^3^, the animals were randomly assigned to different treatment groups such that each group maintained an average tumor size. Animals (*n* = 6) were then treated with either selinexor alone (oral), vMyx-Fluc alone (intratumorally, 2 × 10^7^ FFU on the right tumor only), PBS (oral and intratumoral), or selinexor (oral) plus vMyx-Fluc (intratumorally, 2 × 10^7^ FFU on the right tumor only). Animals received a total of four treatments within the first 2 weeks and the tumor burden was measured two to three times every week. After the first treatment, the mice were imaged using the IVIS system for luciferase expression at 24 and 72 hours posttreatment (Fig. 8B and C). We observed that mice that received selinexor had significantly higher levels of luciferase signals than those injected with the virus alone (Fig. 8C). After imaging and measuring luciferase signals, we provided three additional treatments to test their therapeutic effect on the tumor burden and survival of animals. In addition, we measured body weight for any toxicity to the animals from the treatment. In this PANC-1 xenograft model, we observed a significant reduction in the tumor burden on both sides after treatment with selinexor alone compared with PBS or MYXV-only (Fig. 8D and E). Treatment with selinexor plus MYXV significantly reduced the tumor burden compared with PBS or MYXV-only. However, no significant difference was observed between selinexor and selinexor + MYXV treatments. In this model, we observed an overall reduced tumor burden with the combination treatment of selinexor + MYXV compared with selinexor alone (Supplementary Fig. S3). When we analyzed the survival of animals from different treatment groups, animals treated with selinexor or selinexor + MYXV survived significantly longer than animals treated with either vMyx-Fluc or PBS alone (Fig. 8F). We also observed that animals treated with the combination of selinexor + MYXV survived significantly longer than animals that were treated with selinexor alone.

In addition, we measured luciferase signals in the animals before the endpoint to confirm the presence of the virus in the tumor bed after the last (fourth) injection. Mice injected with MYXV alone (10 days after the last injection) showed high luciferase signals in virus-injected tumors (Supplementary Fig. S4A and S4B). At this point, mice that received selinexor + MYXV also showed a relatively higher level of luciferase signals in the injected tumors than in those treated with MYXV alone. Next, we measured luciferase signals in mice treated with selinexor + MYXV (23 days after the last viral injection) when they reached the endpoint. We observed very high luciferase activity in the injected tumor and very little (one mouse) or no signal in the uninjected tumor (Supplementary Fig. S4C). Finally, we collected both tumors from 3 mice in each group and performed a virus titration assay (Supplementary Fig. S4D). Surprisingly, we detected a lower level of virus in the uninjected tumor, although we did not detect any luciferase signals. Tumors that received selinexor + MYXV than MYXV alone showed a relatively higher level of virus load than the tumors that received only MYXV.

### Both Cellular and Viral Protein Expression Levels are Altered in the Cytoplasmic and Nuclear Compartments after Different Treatments

We performed a global proteome analysis of cytosolic and nuclear compartments to identify the cellular and viral proteins that are changed with different treatments and may contribute to enhanced virus replication, reduced cell proliferation, and cell death. The human colorectal cancer cell line, Colo205, was used for this assay. Samples from mock and those treated with selinexor, MYXV, or a combination of selinexor + MYXV were prepared in quadruple and processed to prepare the nuclear and cytosolic fractions. Approximately 5,000 cellular and viral proteins were identified using mass spectrometry, and their relative abundances in the nuclear and cytosolic fractions were calculated (Supplementary Fig. S5; Supplementary Table S1). We observed the most significant reduction in the abundance of proteins in the nuclear and cytosolic fractions after combining selinexor and MYXV. This was likely due to a substantial decrease in cell proliferation after combination treatment.

## Discussion

Among the many new cancer treatment approaches, OVs have shown tremendous potential in preclinical animal models and clinical trials, allowing the approval of only a few OVs for patients (1). However, there are still limitations to OVs that need to be addressed to obtain more widespread enhanced therapeutic benefits from this treatment approach. One such area of potential development is understanding how OVs and cancer cells interact. The heterogeneity and complexity of the cancer cells in the tumor bed can alter the ability of OVs to replicate in cancer cells. Here, we show for the first time that targeting the nuclear export pathway can enhance the replication of the oncolytic MYXV in typically restricted human cancer cells (defined as either semipermissive or nonpermissive), thereby enhancing its oncolytic ability in preclinical animal models. Like other poxviruses, oncolytic MYXV can promiscuously bind, enter, and initiate infection of most cancer cell types from different tissues and species. However, successful productive replication that leads to progeny virus production and the eventual killing of cancer cells largely depends on the viral manipulation of multiple intracellular signaling pathways (12, 34, 35). For example, several members of the DEAD-box RNA helicases regulate MYXV replication levels in human cancer cells (20). These RNA helicases either inhibit MYXV replication (i.e., antiviral) or are required for optimal virus replication (i.e., proviral). We recently reported that DHX9/RHA forms unique antiviral granules in the cytoplasm, which restrict MYXV replication in human cancer cells by reducing viral late protein synthesis and progeny virus formation (21). DHX9 knockdown in restricted human cancer cells significantly enhanced MYXV gene expression, progeny virus production, cell-to-cell spread, and foci formation (21). Apart from MYXV, DHX9 also has proviral or antiviral roles against diverse RNA and DNA viruses (36, 37).

Similar to many other nuclear RNA helicases, DHX9 shuttles between the nuclear and cytosolic compartments via the classical importin-alpha/beta-dependent pathway to perform diverse cellular functions (38–42). On the basis of these previous reports, we tested nuclear export inhibitors that target XPO1//CRM1 to block the nuclear export of proteins in MYXV-infected human cancer cells. Surprisingly, unlike RNA viruses, blocking the nuclear export pathway using the XPO1 inhibitor LMB in human cancer cells significantly increased MYXV replication, similar to what we observed with the knockdown of DHX9 (21). In addition, LMB treatment significantly reduced the formation of DHX9 antiviral granules in the cytoplasm of the MYXV-infected cells. This observation was also confirmed by knocking down the expression of CRM1 using siRNA. These results suggest that the cellular restriction proteins exported using CRM1 have inhibitory effects on the cytoplasmic replication of MYXV. This is the first report that blocking the CRM1-mediated nuclear export pathway can enhance the replication of any virus and is opposite to what has been reported for many RNA viruses, such as HIV-1, influenza, respiratory syncytial virus, dengue virus, rabies virus, and human cytomegalovirus, all of which depend on the CRM1 nuclear export pathway for replication (43).

Because LMB is relatively toxic to mammalian cells and unsuitable for *in vivo* studies in preclinical animal models, the synthesized derivatives were developed and tested as potential anticancer drugs with minimal toxicity (26, 44). One such LMB derivative, selinexor (KPT330), has been approved by the FDA and is suitable for *in vivo* studies (27). Like LMB, selinexor also significantly enhanced MYXV replication in all restricted human cancer cell lines. This observation was further confirmed by establishing 3D cultures of multiple MYXV-restricted human cancer cell lines. Again, selinexor significantly enhanced viral early and late gene expression compared with MYXV infection alone and greater penetration into the spheroid interior. In addition to enhancing virus production, the combination of selinexor with MYXV significantly reduced cell proliferation and enhanced cancer cell death. More importantly, our results showed that the concentration of selinexor, which has minimal or no toxicity to cells as a single agent, can dramatically increase viral replication and cytotoxicity against cancer cells. These *in vitro* results from the combination of selinexor and MYXV motivated us to test selinexor and MYXV *in vivo* using animal models.

In 2019, the FDA approved selinexor for hematologic malignancies, such as multiple myeloma and lymphoma (27). However, selinexor has also shown promising results against solid tumors, such as lung, breast, pancreatic, melanoma, osteosarcoma, renal, and gastric cancer in preclinical animal models and clinical trials (45–50). A recent study using xenograft animal models of gastric cancer oral delivery of selinexor resulted in significant inhibition of tumor growth (51, 52). In most cancer types, selinexor target XPO1 is overexpressed and correlates with poor clinical outcomes (53). The antitumor activity of selinexor or SINE compounds is achieved by inhibiting cancer cell proliferation by cell-cycle arrest at the G_1_–S phase and inducing apoptosis. Inhibition of XPO1 by selinexor results in nuclear accumulation and functional reactivation of tumor suppressor proteins such as p53, RB1 (retinoblastoma), and CDKN1B (cyclin-dependent kinase inhibitor 1B); reduces translation of oncogene mRNAs such as Myc, Bcl-2 (B-cell lymphoma), and Bcl-6, and NFκB and activation of multiple pathways leading to apoptosis of cancer cells (54). However, in humans, selinexor treatment has shown a reduced number of neutrophils (neutropenia) and white blood cell counts (leukopenia), which may increase the risk of infections. Selinexor also reduces the platelets count (thrombocytopenia), which may cause bleeding (54). These immune modulating effects of selinexor should be addressed by modification of dosages.

Selinexor is delivered orally and thus can be combined with OV delivered either intratumorally or systemically. To test whether selinexor enhances MYXV replication and oncolytic activity *in vivo*, we established a xenograft model using human cancer cells subcutaneously implanted in NSG mice. Our *in vivo* studies with three different MYXV-restricted human cancer cell lines, Colo205, HT29, and PANC-1, demonstrated that oral delivery of selinexor significantly enhanced the replication of MYXV in the tumor bed, as observed by measuring virus-derived luciferase signals *in situ.* Although we used a higher dose (10 mg/kg) in mice than the concentration (between 0.4 and 4.0 mg/kg) of selinexor in cell lines, we observed minimal or no toxicity. A similar dose of selinexor in mice was used in other studies (51, 52). In humans, the recommended starting dosage of selinexor is 80 mg twice per week (160 mg total per week), which can be reduced to 100 or 80 mg, or 60 mg per week depending on the level of adverse reactions. Thus, the concentration of selinexor that enhanced virus replication *in vitro* is within the dose range that is recommended for use in patients (55). Furthermore, the pharmacokinetic studies of selinexor in humans showed peak serum concentration within 2 to 4 hours with a terminal half-life of approximately 6 to 7 hours. There was no evidence of drug accumulation or changes in clearance across different tested dose levels (55).

Selinexor alone significantly reduced the tumor burden bilaterally in all the tested xenograft models compared with the PBS control or MYXV-only treatment. Treatment with selinexor + MYXV also significantly reduced the tumor burden bilaterally compared with the PBS control or MYXV-only treatment. In addition, selinexor + MYXV treatment showed an overall reduced tumor burden than selinexor-only; however, the difference was not statistically significant. Interestingly, in the selinexor + MYXV treatment group, no significant differences were observed between the virus-injected and uninjected tumors. When we measured the virus load from PANC-1 xenograft mice, we observed the presence of MYXV in the uninjected tumor, but only at a very low level. Currently, it is difficult to conclude whether the presence of migrated MYXV, innate immune cells, or a combination of both contributes to this apparent abscopal tumor reduction. Another key finding was that in NSG mice, we observed the persistence of the virus in the injected tumor bed for a relatively prolonged time due to the absence of an active antiviral immune system. This also contributed to the overall reduction in tumor burden in the PANC-1 xenograft model, where selinexor + MYXV treatment significantly enhanced the overall survival of the immunodeficient mice. However, we anticipate that this selinexor + MYXV combination therapy will be more effective in activating antitumor immune responses in the immunocompetent mouse models. Selinexor treatment will enhance virus replication and expression of immune-stimulating transgenes in the tumor bed. For example, engineered MYXV-expressing immune-stimulating cytokines such as TNF or TNF superfamily member 14 (TNFSF14), also known as LIGHT, has shown more therapeutic activity than the wild-type virus (56–58). In addition, enhanced expression of cytokines such as IL15 or IL12 will allow more recruitment of immune cells in the tumor bed (59). We also anticipate that enhanced virus replication will contribute to the more lysis of tumor cells, for example, vMyx-M11L-KO will potentiate antitumor immunity by releasing antigens and activating inflammatory responses in the TME (22). Because MYXV is sensitive to antiviral immune responses in mouse and humans, systemic delivery of MYXV using loaded carrier cells such as mesenchymal stem cells or peripheral blood mononuclear cells provide protection against the antiviral immune responses and enhance the delivery of virus to the tumor bed (57, 60). Thus, based on the current results, the combination of selinexor will further improve the oncolytic activity of MYXV in immunocompetent animal models.

Finally, we performed proteomic analyses of the human colorectal cancer cell line Colo205 after treatment with selinexor, MYXV, and a combination of selinexor and MYXV to determine the global expression level changes in the cellular and viral proteins in the nuclear and cytosolic compartments. Comparing the different treatments and the relative abundance of proteins in the two cellular compartments, we identified both cellular and viral proteins that were upregulated or downregulated by different treatments. At this point, we have been unable to deduce any single pathway responsible for the enhanced anticancer activities of MYXV + selinexor; however, it will be of great interest to further evaluate the function of some specific cellular and viral proteins in the context of virus replication, cell proliferation, and cancer cell death.

## Supplementary Material

Supplementary Table S1 List of proteins identified using mass spectrometry, and their relative abundances in the nuclear and cytosolic fractions.

Supplementary Figure S1 Selinexor enhances MYXV replication in vivo in HT29 xenograft tumors in NSG mice and reduces tumor burden.

Supplementary Figure S2 Tumor burden of individual mouse received Colo205 cells.

Supplementary Figure S3 Tumor burden of individual mouse received PANC-1 cells.

Supplementary Figure S4 Prolonged replication of MYXV in the tumor bed of the Selinexor treated mice.

Supplementary Figure S5 Mass spectrometry of host and viral proteins in the cytoplasmic and nuclear compartments.

## References

[1] Rahman MM , McFadden G. Oncolytic viruses: newest frontier for cancer immunotherapy. Cancers 2021;13:5452. 34771615 10.3390/cancers13215452 PMC8582515

[2] Zhang S , Rabkin SD. The discovery and development of oncolytic viruses: are they the future of cancer immunotherapy? Expert Opin Drug Discov 2021;16:391–410. 33232188 10.1080/17460441.2021.1850689 PMC7969427

[3] Kaufman HL , Kohlhapp FJ, Zloza A. Oncolytic viruses: a new class of immunotherapy drugs. Nat Rev Drug Discov 2015;14:642–62. 26323545 10.1038/nrd4663 PMC7097180

[4] Bell J , McFadden G. Viruses for tumor therapy. Cell Host Microbe 2014;15:260–5. 24629333 10.1016/j.chom.2014.01.002 PMC3963258

[5] Davola ME , Mossman KL. Oncolytic viruses: how “lytic" must they be for therapeutic efficacy? Oncoimmunology 2019;8:e1581528. 10.1080/2162402X.2019.1596006 PMC6492965 31069150

[6] Boagni DA , Ravirala D, Zhang SX. Current strategies in engaging oncolytic viruses with antitumor immunity. Mol Ther Oncolytics 2021;22:98–113. 34514092 10.1016/j.omto.2021.05.002 PMC8411207

[7] Kooti W , Esmaeili Gouvarchin Ghaleh H, Farzanehpour M, Dorostkar R, Jalali Kondori B, Bolandian M. Oncolytic viruses and cancer, do you know the main mechanism? Front Oncol 2021;11:761015. 35004284 10.3389/fonc.2021.761015 PMC8728693

[8] Malfitano AM , Di Somma S, Iannuzzi CA, Pentimalli F, Portella G. Virotherapy: from single agents to combinatorial treatments. Biochem Pharmacol 2020;177:113986. 32330494 10.1016/j.bcp.2020.113986

[9] Zheng N , Fang J, Xue G, Wang Z, Li X, Zhou M, . Induction of tumor cell autosis by myxoma virus-infected CAR-T and TCR-T cells to overcome primary and acquired resistance. Cancer Cell 2022;40:973–985. 36027915 10.1016/j.ccell.2022.08.001 PMC9489043

[10] Zhang Y , Li Y, Chen K, Qian L, Wang P. Oncolytic virotherapy reverses the immunosuppressive tumor microenvironment and its potential in combination with immunotherapy. Cancer Cell Int 2021;21:262. 33985527 10.1186/s12935-021-01972-2 PMC8120729

[11] Malogolovkin A , Gasanov N, Egorov A, Weener M, Ivanov R, Karabelsky A. Combinatorial approaches for cancer treatment using oncolytic viruses: projecting the perspectives through clinical trials outcomes. Viruses 2021;13:1271. 34209981 10.3390/v13071271 PMC8309967

[12] Rahman MM , McFadden G. Oncolytic virotherapy with myxoma virus. J Clin Med 2020;9:171. 31936317 10.3390/jcm9010171 PMC7020043

[13] Chan WM , Rahman MM, McFadden G. Oncolytic myxoma virus: the path to clinic. Vaccine 2013;31:4252–8. 23726825 10.1016/j.vaccine.2013.05.056 PMC3755036

[14] Rahman MM , Madlambayan GJ, Cogle CR, McFadden G. Oncolytic viral purging of leukemic hematopoietic stem and progenitor cells with myxoma virus. Cytokine Growth Factor Rev 2010;21:169–75. 20211576 10.1016/j.cytogfr.2010.02.010 PMC2881168

[15] McFadden G . Poxvirus tropism. Nat Rev Microbiol 2005;3:201–13. 15738948 10.1038/nrmicro1099 PMC4382915

[16] Wang G , Barrett JW, Stanford M, Werden SJ, Johnston JB, Gao X, . Infection of human cancer cells with myxoma virus requires Akt activation via interaction with a viral ankyrin-repeat host range factor. Proc Natl Acad Sci U S A 2006;103:4640–5. 16537421 10.1073/pnas.0509341103 PMC1450224

[17] Rahman MM , Liu J, Chan WM, Rothenburg S, McFadden G. Myxoma virus protein M029 is a dual function immunomodulator that inhibits PKR and also conscripts RHA/DHX9 to promote expanded host tropism and viral replication. PLoS Pathog 2013;9:e1003465. 23853588 10.1371/journal.ppat.1003465 PMC3701710

[18] Kim M , Williamson CT, Prudhomme J, Bebb DG, Riabowol K, Lee PWK, . The viral tropism of two distinct oncolytic viruses, reovirus and myxoma virus, is modulated by cellular tumor suppressor gene status. Oncogene 2010;29:3990–6. 20473328 10.1038/onc.2010.137 PMC4374435

[19] Bartee E , McFadden G. Human cancer cells have specifically lost the ability to induce the synergistic state caused by tumor necrosis factor plus interferon-beta. Cytokine 2009;47:199–205. 19640730 10.1016/j.cyto.2009.06.006 PMC4376283

[20] Rahman MM , Bagdassarian E, Ali MAM, McFadden G. Identification of host DEAD-box RNA helicases that regulate cellular tropism of oncolytic myxoma virus in human cancer cells. Sci Rep 2017;7:15710. 29146961 10.1038/s41598-017-15941-1 PMC5691082

[21] Rahman MM , Gutierrez-Jensen AD, Glenn HL, Abrantes M, Moussatche N, McFadden G. RNA Helicase A/DHX9 forms unique cytoplasmic antiviral granules that restrict oncolytic myxoma virus replication in human cancer cells. J Virol 2021;95:e0015121. 33952639 10.1128/JVI.00151-21 PMC8223942

[22] Pisklakova A , McKenzie B, Zemp F, Lun X, Kenchappa RS, Etame AB, . M011L-deficient oncolytic myxoma virus induces apoptosis in brain tumor-initiating cells and enhances survival in a novel immunocompetent mouse model of glioblastoma. Neuro Oncol 2016;18:1088-98. 26962017 10.1093/neuonc/now006 PMC4933479

[23] Smallwood SE , Rahman MM, Smith DW, McFadden G. Myxoma virus: propagation, purification, quantification, and storage. Curr Protoc Microbiol 2010;Chapter 14:Unit 14A.1. 10.1002/9780471729259.mc14a01s17 PMC2901999 20440681

[24] Mor A , White MA, Fontoura BMA. Nuclear trafficking in health and disease. Curr Opin Cell Biol 2014;28:28–35. 24530809 10.1016/j.ceb.2014.01.007 PMC4061247

[25] Sloan KE , Gleizes PE, Bohnsack MT. Nucleocytoplasmic transport of RNAs and RNA-protein complexes. J Mol Biol 2016;428:2040–59. 26434509 10.1016/j.jmb.2015.09.023

[26] Mutka SC , Yang WQ, Dong SD, Ward SL, Craig DA, Timmermans PBMWM, . Identification of nuclear export inhibitors with potent anticancer activity *in vivo*. Cancer Res 2009;69:510–7. 19147564 10.1158/0008-5472.CAN-08-0858 PMC2635062

[27] Richard S , Richter J, Jagannath S. Selinexor: a first-in-class SINE compound for treatment of relapsed refractory multiple myeloma. Future Oncol 2020;16:1331–50. 32511022 10.2217/fon-2020-0054

[28] Nachmias B , Schimmer AD. Targeting nuclear import and export in hematological malignancies. Leukemia 2020;34:2875–86. 32624581 10.1038/s41375-020-0958-y PMC7584478

[29] Zheng Y , Gery S, Sun H, Shacham S, Kauffman M, Koeffler HP. KPT-330 inhibitor of XPO1-mediated nuclear export has anti-proliferative activity in hepatocellular carcinoma. Cancer Chemother Pharmacol 2014;74:487–95. 25030088 10.1007/s00280-014-2495-8 PMC4146741

[30] Johnston JB , Wang G, Barrett JW, Nazarian SH, Colwill K, Moran M, . Myxoma virus M-T5 protects infected cells from the stress of cell cycle arrest through its interaction with host cell cullin-1. J Virol 2005;79:10750–63. 16051867 10.1128/JVI.79.16.10750-10763.2005 PMC1182661

[31] Chehrehasa F , Meedeniya ACB, Dwyer P, Abrahamsen G, Mackay-Sim A. EdU, a new thymidine analogue for labelling proliferating cells in the nervous system. J Neurosci Methods 2009;177:122–30. 18996411 10.1016/j.jneumeth.2008.10.006

[32] Everett H , Barry M, Sun X, Lee SF, Frantz C, Berthiaume LG, . The myxoma poxvirus protein, M11L, prevents apoptosis by direct interaction with the mitochondrial permeability transition pore. J Exp Med 2002;196:1127–39. 12417624 10.1084/jem.20011247 PMC2194110

[33] Lv D , Hu Z, Lu L, Lu H, Xu X. Three-dimensional cell culture: a powerful tool in tumor research and drug discovery. Oncol Lett 2017;14:6999–7010. 29344128 10.3892/ol.2017.7134 PMC5754907

[34] Rahman MM , McFadden G. Myxoma virus-encoded host range protein M029: a multifunctional antagonist targeting multiple host antiviral and innate immune pathways. Vaccines 2020;8:244. 32456120 10.3390/vaccines8020244 PMC7349962

[35] Werden SJ , Rahman MM, McFadden G. Poxvirus host range genes. Adv Virus Res 2008;71:135–71. 18585528 10.1016/S0065-3527(08)00003-1

[36] Ullah R , Li J, Fang P, Xiao S, Fang L. DEAD/H-box helicases: anti-viral and pro-viral roles during infections. Virus Res 2022;309:198658. 34929216 10.1016/j.virusres.2021.198658

[37] Guo F , Xing L. RNA helicase A as co-factor for DNA viruses during replication. Virus Res 2021;291:198206. 33132162 10.1016/j.virusres.2020.198206

[38] Tang H , McDonald D, Middlesworth T, Hope TJ, Wong-Staal F. The carboxyl terminus of RNA helicase A contains a bidirectional nuclear transport domain. Mol Cell Biol 1999;19:3540–50. 10207077 10.1128/mcb.19.5.3540 PMC84146

[39] Fujita H , Ohshima T, Oishi T, Aratani S, Fujii R, Fukamizu A, . Relevance of nuclear localization and functions of RNA helicase A. Int J Mol Med 2005;15:555–60. 15754013

[40] Aratani S , Oishi T, Fujita H, Nakazawa M, Fujii R, Imamoto N, . The nuclear import of RNA helicase A is mediated by importin-alpha3. Biochem Biophys Res Commun 2006;340:125–33. 16375861 10.1016/j.bbrc.2005.11.161

[41] Jefferson M , Donaszi-Ivanov A, Pollen S, Dalmay T, Saalbach G, Powell PP. Host factors that interact with the pestivirus N-terminal protease, Npro, are components of the ribonucleoprotein complex. J Virol 2014;88:10340–53. 24965446 10.1128/JVI.00984-14 PMC4178888

[42] Liu L , Tian J, Nan H, Tian M, Li Y, Xu X, . Porcine reproductive and respiratory syndrome virus nucleocapsid protein interacts with Nsp9 and cellular DHX9 to regulate viral RNA synthesis. J Virol 2016;90:5384–98. 27009951 10.1128/JVI.03216-15 PMC4934760

[43] Mathew C , Ghildyal R. CRM1 inhibitors for antiviral therapy. Front Microbiol 2017;8:1171. 28702009 10.3389/fmicb.2017.01171 PMC5487384

[44] Newlands ES , Rustin GJ, Brampton MH. Phase I trial of elactocin. Br J Cancer 1996;74:648–9. 8761384 10.1038/bjc.1996.415 PMC2074658

[45] Ho J , Heong V, Peng Yong W, Soo R, Ean Chee C, Wong A, . A phase 1 study of the safety, pharmacokinetics and pharmacodynamics of escalating doses followed by dose expansion of the selective inhibitor of nuclear export (SINE) selinexor in Asian patients with advanced or metastatic malignancies. Ther Adv Med Oncol 2022;14:17588359221087555. 35432603 10.1177/17588359221087555 PMC9008867

[46] Landes JR , Moore SA, Bartley BR, Doan HQ, Rady PL, Tyring SK. The efficacy of selinexor (KPT-330), an XPO1 inhibitor, on non-hematologic cancers: a comprehensive review. J Cancer Res Clin Oncol 2023;149:2139–55. 35941226 10.1007/s00432-022-04247-z PMC11796554

[47] Thirasastr P , Somaiah N. Overview of systemic therapy options in liposarcoma, with a focus on the activity of selinexor, a selective inhibitor of nuclear export in dedifferentiated liposarcoma. Ther Adv Med Oncol 2022;14:17588359221081073. 35251319 10.1177/17588359221081073 PMC8891917

[48] Arango NP , Yuca E, Zhao M, Evans KW, Scott S, Kim C, . Selinexor (KPT-330) demonstrates anti-tumor efficacy in preclinical models of triple-negative breast cancer. Breast Cancer Res 2017;19:93. 28810913 10.1186/s13058-017-0878-6 PMC5557476

[49] Rosen JC , Weiss J, Pham NA, Li Q, Martins-Filho SN, Wang Y, . Antitumor efficacy of XPO1 inhibitor Selinexor in KRAS-mutant lung adenocarcinoma patient-derived xenografts. Transl Oncol 2021;14:101179. 34284202 10.1016/j.tranon.2021.101179 PMC8313753

[50] Kazim S , Malafa MP, Coppola D, Husain K, Zibadi S, Kashyap T, . Selective nuclear export inhibitor KPT-330 enhances the antitumor activity of gemcitabine in human pancreatic cancer. Mol Cancer Ther 2015;14:1570–81. 25934708 10.1158/1535-7163.MCT-15-0104 PMC4577050

[51] Subhash VV , Yeo MS, Wang L, Tan SH, Wong FY, Thuya WL, . Anti-tumor efficacy of Selinexor (KPT-330) in gastric cancer is dependent on nuclear accumulation of p53 tumor suppressor. Sci Rep 2018;8:12248. 30115935 10.1038/s41598-018-30686-1 PMC6095850

[52] Sexton R , Mahdi Z, Chaudhury R, Beydoun R, Aboukameel A, Khan HY, . Targeting nuclear exporter protein XPO1/CRM1 in gastric cancer. Int J Mol Sci 2019;20:4826. 31569391 10.3390/ijms20194826 PMC6801932

[53] Marretta AL , Di Lorenzo G, Ribera D, Cannella L, von Arx C, Bracigliano A, . Selinexor and the selective inhibition of nuclear export: a new perspective on the treatment of sarcomas and other solid and non-solid tumors. Pharmaceutics 2021;13:1522. 34575598 10.3390/pharmaceutics13091522 PMC8466603

[54] Sellin M , Berg S, Hagen P, Zhang J. The molecular mechanism and challenge of targeting XPO1 in treatment of relapsed and refractory myeloma. Transl Oncol 2022;22:101448. 35660848 10.1016/j.tranon.2022.101448 PMC9166471

[55] Abdul Razak AR , Mau-Soerensen M, Gabrail NY, Gerecitano JF, Shields AF, Unger TJ, . First-in-class, first-in-human phase I study of selinexor, a selective inhibitor of nuclear export, in patients with advanced solid tumors. J Clin Oncol 2016;34:4142–50. 26926685 10.1200/JCO.2015.65.3949 PMC5562433

[56] Christie JD , Appel N, Canter H, Achi JG, Elliott NM, de Matos AL, . Systemic delivery of TNF-armed myxoma virus plus immune checkpoint inhibitor eliminates lung metastatic mouse osteosarcoma. Mol Ther Oncolytics 2021;22:539–54. 34553039 10.1016/j.omto.2021.07.014 PMC8433070

[57] Christie JD , Appel N, Zhang L, Lowe K, Kilbourne J, Daggett-Vondras J, . Systemic delivery of mLIGHT-armed myxoma virus is therapeutic for later-stage syngeneic murine lung metastatic osteosarcoma. Cancers 2022;14:337. 35053501 10.3390/cancers14020337 PMC8773855

[58] Jazowiecka-Rakus J , Hadrys A, Rahman MM, McFadden G, Fidyk W, Chmielik E, . Myxoma virus expressing light (TNFSF14) pre-loaded into adipose-derived mesenchymal stem cells is effective treatment for murine pancreatic adenocarcinoma. Cancers 2021;13:1394. 33808692 10.3390/cancers13061394 PMC8003548

[59] Tosic V , Thomas DL, Kranz DM, Liu J, McFadden G, Shisler JL, . Myxoma virus expressing a fusion protein of interleukin-15 (IL15) and IL15 receptor alpha has enhanced antitumor activity. PLoS One 2014;9:e109801. 25329832 10.1371/journal.pone.0109801 PMC4199602

[60] Jazowiecka-Rakus J , Sochanik A, Rusin A, Hadryś A, Fidyk W, Villa N, . Myxoma virus-loaded mesenchymal stem cells in experimental oncolytic therapy of murine pulmonary melanoma. Mol Ther Oncolytics 2020;18:335–50. 32775618 10.1016/j.omto.2020.07.003 PMC7398944

