# Supplementary material for: Nuclear Export Inhibitor Selinexor Enhances Oncolytic Myxoma Virus Therapy against Cancer
Source: Cancer Res Commun. 2023 Jun 1;3(6):952–68. doi: 10.1158/2767-9764.CRC-22-0483 (PMC10234290; doi:10.1158/2767-9764.CRC-22-0483)
Supplement: Supplementary Figure S1 — Selinexor enhances MYXV replication in vivo in HT29 xenograft tumors in NSG mice and reduces tumor burden. [file crc-22-0483-s02.pptx]

## Slide 1
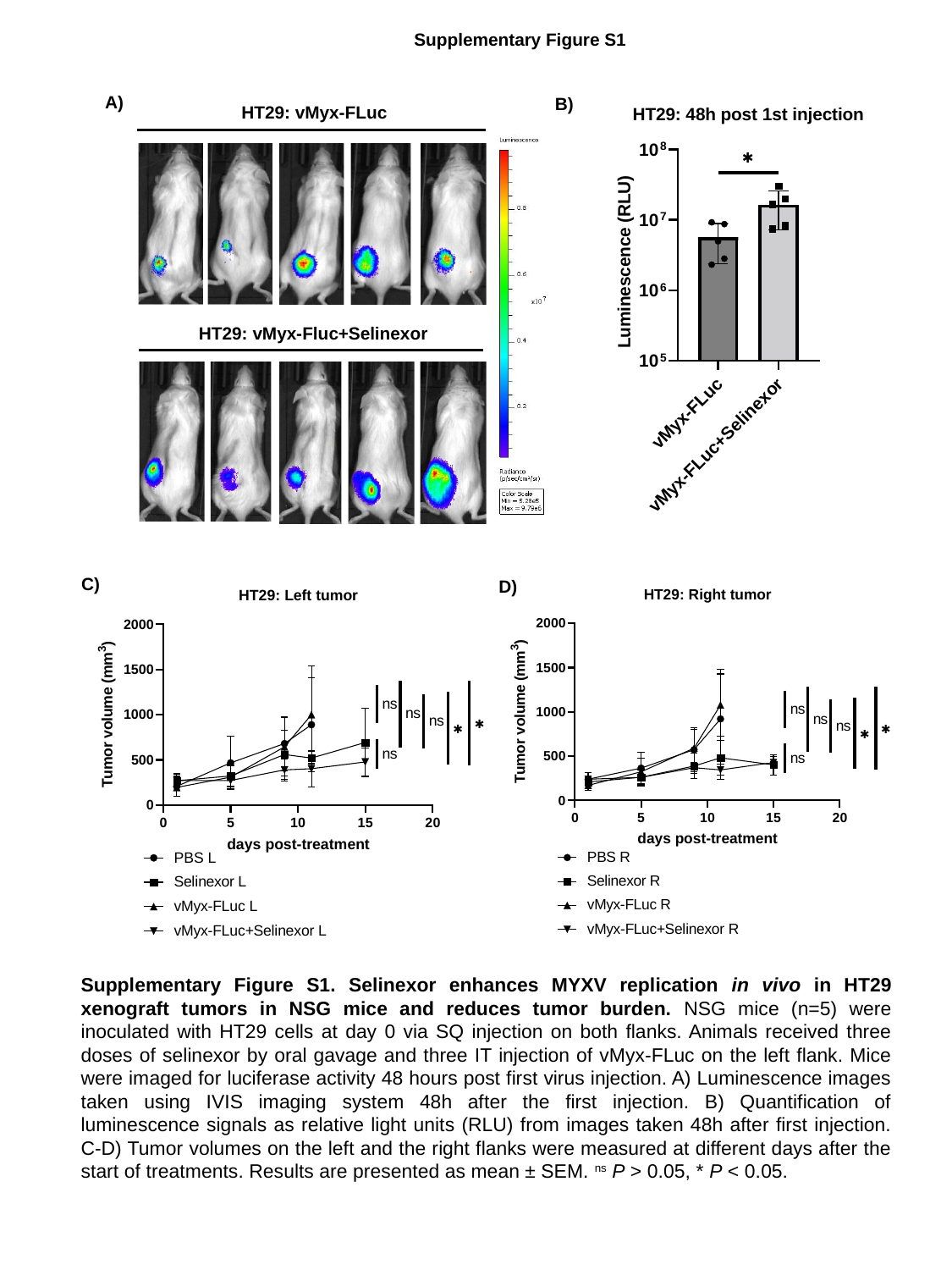

Supplementary Figure S1
A)
B)
HT29: vMyx-FLuc
HT29: vMyx-Fluc+Selinexor
C)
D)
Supplementary Figure S1. Selinexor enhances MYXV replication in vivo in HT29 xenograft tumors in NSG mice and reduces tumor burden. NSG mice (n=5) were inoculated with HT29 cells at day 0 via SQ injection on both flanks. Animals received three doses of selinexor by oral gavage and three IT injection of vMyx-FLuc on the left flank. Mice were imaged for luciferase activity 48 hours post first virus injection. A) Luminescence images taken using IVIS imaging system 48h after the first injection. B) Quantification of luminescence signals as relative light units (RLU) from images taken 48h after first injection. C-D) Tumor volumes on the left and the right flanks were measured at different days after the start of treatments. Results are presented as mean ± SEM. ns P > 0.05, * P < 0.05.
