# Supplementary material for: Nuclear Export Inhibitor Selinexor Enhances Oncolytic Myxoma Virus Therapy against Cancer
Source: Cancer Res Commun. 2023 Jun 1;3(6):952–68. doi: 10.1158/2767-9764.CRC-22-0483 (PMC10234290; doi:10.1158/2767-9764.CRC-22-0483)
Supplement: Supplementary Figure S2 — Tumor burden of individual mouse received Colo205 cells. [file crc-22-0483-s03.pptx]

## Slide 1
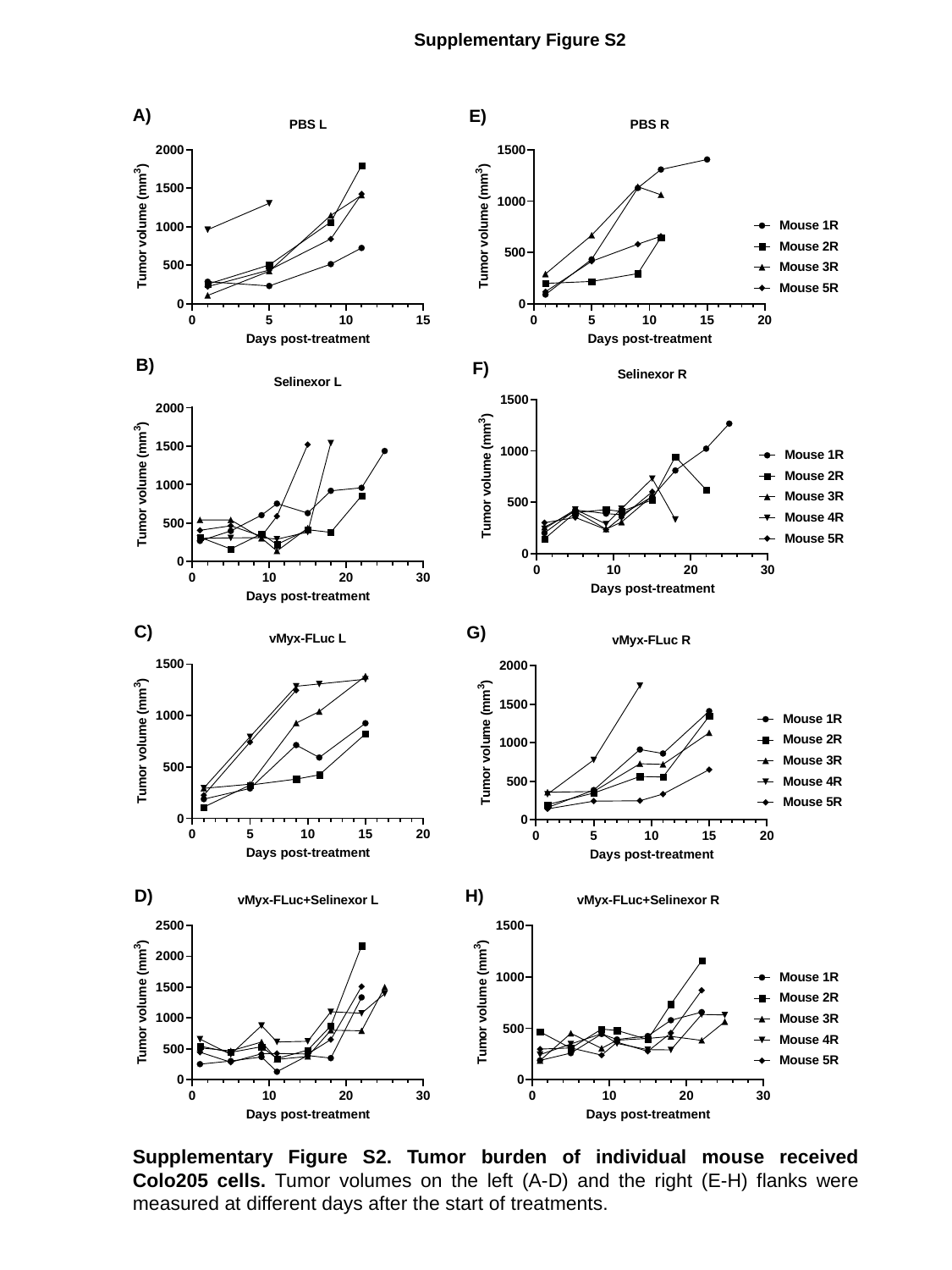

Supplementary Figure S2
A)
E)
B)
F)
C)
G)
D)
H)
Supplementary Figure S2. Tumor burden of individual mouse received Colo205 cells. Tumor volumes on the left (A-D) and the right (E-H) flanks were measured at different days after the start of treatments.
