# Supplementary material for: Nuclear Export Inhibitor Selinexor Enhances Oncolytic Myxoma Virus Therapy against Cancer
Source: Cancer Res Commun. 2023 Jun 1;3(6):952–68. doi: 10.1158/2767-9764.CRC-22-0483 (PMC10234290; doi:10.1158/2767-9764.CRC-22-0483)
Supplement: Supplementary Figure S4 — Prolonged replication of MYXV in the tumor bed of the Selinexor treated mice. [file crc-22-0483-s05.pptx]

## Slide 1
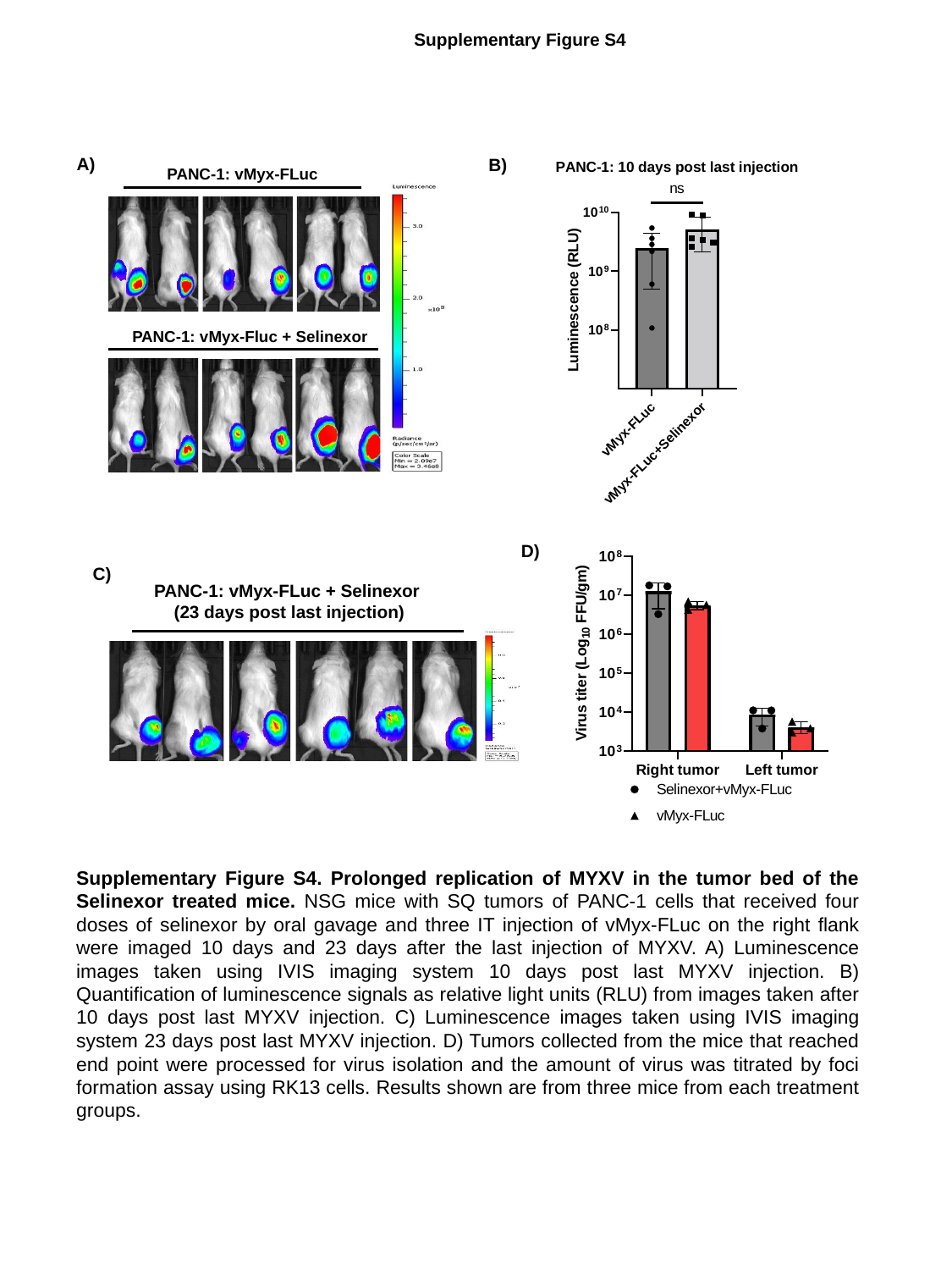

Supplementary Figure S4
A)
B)
PANC-1: vMyx-FLuc
PANC-1: vMyx-Fluc + Selinexor
D)
C)
PANC-1: vMyx-FLuc + Selinexor
(23 days post last injection)
Supplementary Figure S4. Prolonged replication of MYXV in the tumor bed of the Selinexor treated mice. NSG mice with SQ tumors of PANC-1 cells that received four doses of selinexor by oral gavage and three IT injection of vMyx-FLuc on the right flank were imaged 10 days and 23 days after the last injection of MYXV. A) Luminescence images taken using IVIS imaging system 10 days post last MYXV injection. B) Quantification of luminescence signals as relative light units (RLU) from images taken after 10 days post last MYXV injection. C) Luminescence images taken using IVIS imaging system 23 days post last MYXV injection. D) Tumors collected from the mice that reached end point were processed for virus isolation and the amount of virus was titrated by foci formation assay using RK13 cells. Results shown are from three mice from each treatment groups.
