# Supplementary material for: Nuclear Export Inhibitor Selinexor Enhances Oncolytic Myxoma Virus Therapy against Cancer
Source: Cancer Res Commun. 2023 Jun 1;3(6):952–68. doi: 10.1158/2767-9764.CRC-22-0483 (PMC10234290; doi:10.1158/2767-9764.CRC-22-0483)
Supplement: Supplementary Figure S5 — Mass spectrometry of host and viral proteins in the cytoplasmic and nuclear compartments. [file crc-22-0483-s06.pptx]

## Slide 1
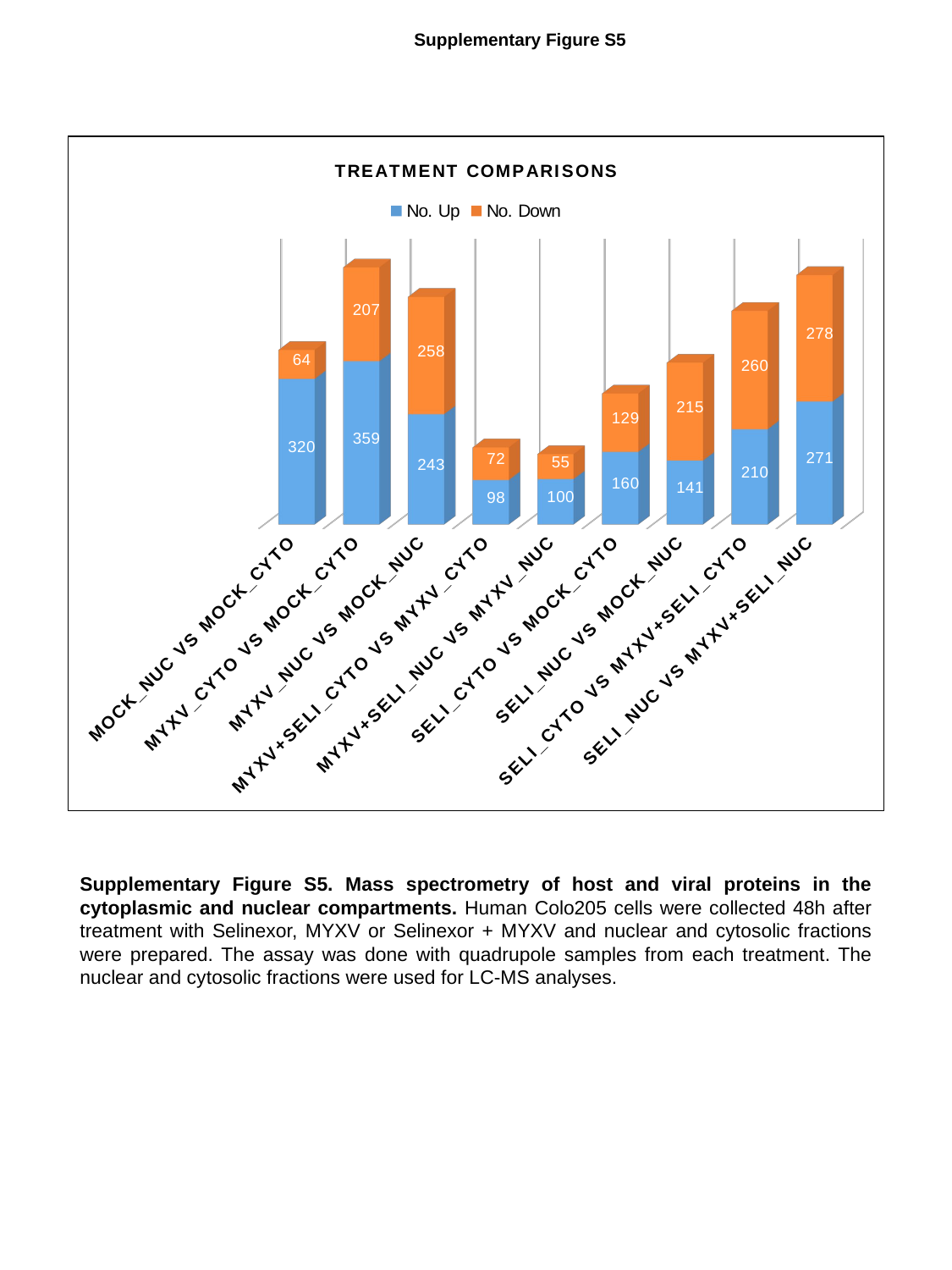

Supplementary Figure S5
[unsupported chart]
Supplementary Figure S5. Mass spectrometry of host and viral proteins in the cytoplasmic and nuclear compartments. Human Colo205 cells were collected 48h after treatment with Selinexor, MYXV or Selinexor + MYXV and nuclear and cytosolic fractions were prepared. The assay was done with quadrupole samples from each treatment. The nuclear and cytosolic fractions were used for LC-MS analyses.
